# Multicomponent polysaccharide–protein bioconjugation in the development of antibacterial glycoconjugate vaccine candidates†
†Electronic supplementary information (ESI) available. See DOI: 10.1039/c7sc05467j


**DOI:** 10.1039/c7sc05467j

**Published:** 2018-01-19

**Authors:** Yanira Méndez, Janoi Chang, Ana R. Humpierre, Abel Zanuy, Raine Garrido, Aldrin V. Vasco, Jessy Pedroso, Darielys Santana, Laura M. Rodríguez, Dagmar García-Rivera, Yury Valdés, Vicente Vérez-Bencomo, Daniel G. Rivera

**Affiliations:** a Center for Natural Products Research , Faculty of Chemistry , University of Havana , Zapata y G , Havana 10400 , Cuba . Email: dgr@fq.uh.cu; b Finlay Institute of Vaccines , Ave 27 Nr. 19805 , Havana 10600 , Cuba . Email: vicente.verez@finlay.edu.cu; c Department of Bioorganic Chemistry , Leibniz Institute of Plant Biochemistry , Weinberg 3 , 06120 , Halle/Saale , Germany

## Abstract

A new synthetic strategy for the development of multivalent antibacterial glycoconjugate vaccines is described. The approach comprises the utilization of an isocyanide-based multicomponent process for the conjugation of functionalized capsular polysaccharides of *S. pneumoniae* and *S.* Typhi to carrier proteins such as diphtheria and tetanus toxoids. For the first time, oxo- and carboxylic acid-functionalized polysaccharides could be either independently or simultaneously conjugated to immunogenic proteins by means of the Ugi-multicomponent reaction, thus leading to mono- or multivalent unimolecular glycoconjugates as vaccine candidates. Despite the high molecular weight of the two or three reacting biomolecules, the multicomponent bioconjugation proved highly efficient and reproducible. The Ugi-derived glycoconjugates showed notable antigenicity and elicited good titers of functional specific antibodies. To our knowledge, this is the only bioconjugation method that enables the incorporation of two different polysaccharidic antigens to a carrier protein in a single step. Applications in the field of self-adjuvanting, eventually anticancer, multicomponent vaccines are foreseeable.

## Abstract 2

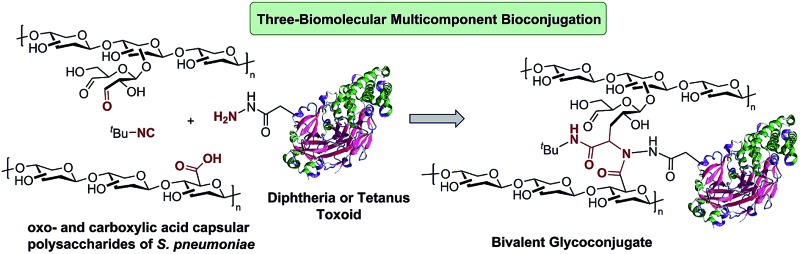
A multicomponent reaction enables the one-pot assembly of immunogenic multivalent glycoconjugates.

## Introduction

Multicomponent reactions (MCRs) are convergent processes in which three or more reactants are combined in one pot to render a product that incorporates atoms from all starting materials.^1^ Owing to their high chemical efficiency, diversity and complexity-generating character,^2^ these processes have been widely exploited in drug discovery^3^ and natural product synthesis.^4^ Remarkable applications of MCRs are also found in heterocyclic,^5^ cyclic peptide^6^ and carbohydrate chemistry.^7^ However, MCRs have been rarely exploited in protein bioconjugation^8^,^9^ and, to our knowledge, they have never been employed in the development of glycoconjugate vaccines or in immunological applications.

Among the MCRs, the Ugi four-component reaction stands out as one of the most powerful approaches to produce biologically relevant molecules.^10^ This procedure, which comprises the efficient condensation of a primary amine, a carboxylic acid, an oxo-compound and an isocyanide, has been previously used for the conjugation of oligo- and polysaccharides to other molecules.^11^ This reaction has been seldom employed in the covalent modification of proteins, such as bovine serum albumin (BSA), horseradish peroxidase (HRP) and bovine pancreatic trypsin,^9^ but not in such a way that several saccharidic components are simultaneously conjugated to a protein.

As part of our effort to develop synthetic methods toward antibacterial conjugate vaccines,^12^ herein we report an innovative application of MCRs in the fields of glycobiology and bioconjugation. The strategy encompasses the utilization of the Ugi reaction as an effective method for the conjugation of either one or two bacterial capsular polysaccharides (CPs) to carrier proteins, thus leading to uni- or bivalent glycoconjugates. The scope of this multicomponent bioconjugation is assessed by employing oxo- and carboxylic acid-functionalized CPs of different bacteria or serotypes as well as tetanus toxoid (TT) and diphtheria toxoid (DT) as the amino components of the process, along with commercial isocyanides. This work represents the first endeavor toward the development of glycoconjugate vaccine candidates by means of MCRs.

The immunogenic role of bacterial CPs and their importance in the development of prophylactic vaccines have been known since the beginning of the last century.^13^ Although the advent of antibiotics discouraged the application of antibacterial carbohydrate-based vaccines, the emergence of resistance against antibiotics has led to a renaissance of this strategy.^14^ Owing to their T-cell independent response, carbohydrate-based vaccines provide only short term protection for most bacterial infections, being of limited value in high risk groups such as infants and immuno-compromised people.^15^ Proteins, on the other hand, are T-cell dependent antigens with a resulting long lasting immunization. The conjugation of CPs to carrier proteins is known to enhance the immunogenicity when compared with the natural polysaccharide.^16^

Several methods have been established for the synthesis of glycoproteins and glycoconjugate vaccines, including classical reductive amination, peptide coupling and maleimide-mediated conjugation,^17^ as well as new site-specific methods such as alkyne–azide dipolar cycloaddition^18^ and thioether formation,^19^ among others.^20^

## Results and discussion

An advantage of using the Ugi reaction in the conjugation step is that many of the Ugi-reactive functional groups are either already present in the protein (*e.g.*, carboxylic acid and amine) or are easy to introduce into the polysaccharide (*e.g.*, aldehyde and carboxylic acid) by a variety of known procedures.^9^,^11^ Another feature, which favors the utilization of this multicomponent process is the smooth implementation of the reaction setup under standard bioconjugation conditions, *i.e.*, room temperature, non-inert atmosphere, aqueous conditions, *etc.*^21^ Our initial strategy focused on the development of glycoconjugate vaccine candidates of different serotypes of *Streptococcus pneumoniae*.

Bacterial infections caused by this pathogen constitute the first cause of pneumonia, meningitis, sepsis and non-acute otitis in the world.^22^ They are also the first cause of death by preventable diseases in children under five years.^23^ Among the different *S. pneumoniae* serotypes of global incidence, the CPs of serotypes 14 (CPs14), 9V (CPs9V) and 7F (CPs7F) were chosen at first and subjected to a controlled periodic oxidation,^24^ aimed at generating the carbonyl groups (1 carbonyl every 6 or 7 repetitive units) required for the Ugi reaction without altering their antigenic determinants (see the ESI†). TT and DT were selected as carrier proteins, as they have shown great efficacy in vaccination strategies worldwide.^25^ These proteins were produced in-house and then detoxified according to production procedures recommended by the World Health Organization (WHO).^26^ Both proteins were next subjected to hydrazide activation by the controlled reaction of hydrazine with aspartic and glutamic acid side chains (see the ESI†), aimed at introducing more reactive amino groups. As oxo-functionalized CPs have never been conjugated to such large proteins by means of isocyanide-MCRs, we initially focused on assessing the scope of the multicomponent conjugation protocol and the immunogenicity of the Ugi-derived glycoconjugates.

**Scheme 1:**
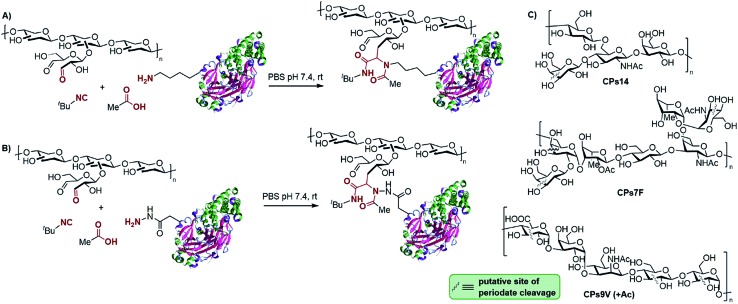
Multicomponent bioconjugation of oxo-functionalized CPs (putative truncated fragments are shown to represent the repetitive units of CPs) of *S. pneumoniae* to (A) non-activated DT and TT and (B) hydrazide-activated DT and TT. (C) Repetitive units of the CPs of *S. pneumoniae* serotypes 14, 7F and 9V, used as oxo-components after periodic oxidation.

The conjugation of the oxo-functionalized CPs of the three *S. pneumoniae* serotypes (Scheme 1A, shown as putative generic structures) was first carried out for non-activated DT and TT. Acetic acid and *tert*-butyl isocyanide were chosen as the other components of the Ugi reaction as they represent the smallest reagents available with the hope of avoiding the incorporation of new immunogenic moieties (smaller isocyanides are too volatile and smelly). The multicomponent protocol comprises the addition of an excess of the carboxylic acid and isocyanide components to a 1 : 1 (w/w) stirring mixture of the protein and polysaccharide. Scheme 1A depicts a generic representation of the linkage between both macromolecules, although the attachment sites cannot be actually known, as more complex structures with cross-linked lattice matrixes could be formed. Although MCRs are generally favored at higher concentrations, conjugations were performed either at 5 mg mL^–1^ or 10 mg mL^–1^ in PBS at pH 7.4 to avoid gelification of the polysaccharides. Analysis of the glycoconjugates was accomplished by following the suggestions and the most relevant requirements of the WHO guidelines for producing conjugate vaccines, which recommend size exclusion HPLC (SE-HPLC) and Nuclear Magnetic Resonance (NMR) for this type of polysaccharide–protein conjugate.^27^ Thus, the identity of the polysaccharide epitope in the glycoconjugates was evaluated using proton NMR to confirm the fingerprint of each polysaccharide involved (see the ESI†). It must be noted that because the CPs used are polydisperse macromolecules (*i.e.*, 10–100 kDa), the utilization of mass spectrometry is not as valuable as it is in the case of glycoconjugates derived from synthetic oligosaccharidic antigens.

Initially, the efficiency of the conjugation of CPs14 to non-activated TT in the presence of acetic acid and *t*-butyl isocyanide at 0 h, 4 h, 24 h and 48 h, was monitored using SE-HPLC. Thus, about 43% of non-conjugated protein persisted after 4 h, a value that remained equally high (*ca.* 41%) after 24 h and 48 h of reaction. Similar results were obtained for the conjugation of the CPs of serotype 14 to non-activated DT and BSA (see the ESI†). We hypothesize that inefficient imine formation between the protein and the oxo-functionalized CPs would be a reason for the poor conjugation efficiency. Indeed, this is a crucial step not only for the Ugi reaction, but also for conjugation protocols based on reductive amination. This may be due to the low number and poor accessibility of lysine side-chain amino groups after detoxification of the native toxin. As a result, we envisioned that the hydrazide-activated toxoids would lead to more effective imine formation and, consequently, to a faster Ugi reaction, due to the greater nucleophilicity of hydrazides compared to amines.

The multicomponent conjugation of hydrazide-activated TT (TT^a^) to the oxo-functionalized CPs14 was undertaken also in the presence of acetic acid and *t*-butyl isocyanide (Scheme 1B). Remarkably, the reaction was completed in only 4 h, as demonstrated by SE-HPLC. A similar result was obtained for the conjugation of oxo-functionalized CPs14 to hydrazide-activated DT (DT^a^). Formation of the glycoconjugate was also confirmed by sodium dodecylsulfate polyacrylamide gel electrophoresis (SDS-PAGE), with a specific staining for the protein (Coomassie) and the carbohydrate (Fuchsin), where the presence of a carbohydrate–protein conjugate of higher molecular weight than the free protein was corroborated (see the ESI†). After this initial result, we sought to broaden the scope of this conjugation protocol with the other *S. pneumoniae* CPs. Gratifyingly, the multicomponent bioconjugation of oxo-functionalized CPs9V and CPs7F to both TT^a^ and DT^a^ also proved highly efficient, which confirms the reproducibility of this method to produce glycoconjugates. Based on our own experience of the production of conjugate vaccines in clinical use,^12^ we can confirm that both the efficiency and viability of the experimental setups are similar to other standard conjugation methods, such as reductive amination and Michael addition to maleimides.

All glycoconjugates were purified by diafiltration through a regenerated cellulose membrane (100 kDa) and characterized by SE-HPLC and colorimetric techniques. The free protein percentages (FPPs) and the CP/protein ratios were determined for each conjugate (see Table S2 in the ESI†). In all cases, the FPPs were below 20% (and sometimes null) and the CP/protein ratios were between 1 : 2 and 2 : 1, which are within the range for pneumococcal conjugate vaccines (PVC) recommended by the WHO (*i.e.*, 1 : 3 to 3 : 1).^27^ The stability of the conjugates in solution was periodically monitored over 3 months by employing Superose® 12 and TSK 5000 SE-HPLCs. To our delight, no appreciable changes were observed within this period of time.

**Fig. 1:**
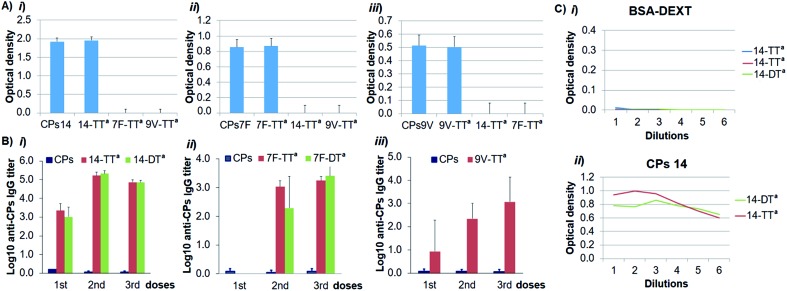
(A) Antigenicity evaluation of conjugates 14-TT^a^, 7F-TT^a^ and 9V-TT^a^ by ELISA with: (i) a specific anti-CPs14 antibody, (ii) a specific anti-CPs7F antibody and (iii) a specific anti-CPs9V antibody. (B) Anti-CPs IgG titers elicited in mice by the glycoconjugates, as determined by ELISA: (i) 14-TT^a^ and 14-DT^a^, (ii) 7F-TT^a^ and 7F-DT^a^ and (iii) 9V-TT^a^. (C) Assessment of the presence, in the bleed-sera, of specific antibodies against: (i) an Ugi-derived BSA–dextran conjugate and (ii) the CPs14. The scale 1 to 6 is a serial dilution (1/2) from 1 : 100 to 1 : 3200.

To demonstrate that the antigenicity on the conjugated CPs was preserved, we carried out an indirect enzyme-linked immunosorbent assay (ELISA) with the corresponding Ugi-derived glycoconjugates, the natural CPs as a reference and their specific antibodies. As depicted in Fig. 1A, the recognition of each glycoconjugate by its specific antibody was similar to that of the natural CPs, which confirms that the antigenic determinant integrity was conserved in the glycoconjugate structures. We next turned to evaluate the immunogenicity of the glycoconjugates by immunization of Balb/c mice. The specific IgG induced response was determined by ELISA and the antibody titers against the CPs were defined as the logarithms of the highest serum dilution giving two-fold the absorbance value of pre-immune sera. The IgG titers elicited by each Ugi-derived glycoconjugate – adsorbed on AlPO_4_ – showed a relevant increase compared with those of the natural CPs (Fig. 1B). This result indicates that the glycoconjugates are able to induce a T-dependent response against the CPs, and therefore, they could be considered as valuable vaccine candidates.

As this multicomponent conjugation introduces a peptidic linkage between the two biomolecules that includes the non-natural *t*-butyl group, we considered the possibility of such an Ugi-linkage to be independently recognized by the immune system. To assess whether anti-Ugi-linkage antibodies could be elicited, fragmented oxidized-dextran was conjugated to hydrazide-activated BSA following the same Ugi-conjugation protocol and the resulting glycoconjugate was analyzed by ELISA. For this, the microtiter plate was coated with the dextran–BSA Ugi-derived conjugate and serial dilutions of final bleed-sera from the immunization with the conjugates CPs14-TT^a^ and CPs14-DT^a^ were added. A parallel ELISA experiment was conducted with the natural CPs14 as a positive control. As depicted in Fig. 1C, the bleed-sera do not contain specific antibodies against either the Ugi-linkage or, as expected, the dextran and BSA antigens, while the positive control shows specific recognition of the natural CPs14 by the bleed-sera antibodies. This fact demonstrates that the Ugi-linkage is non-immunogenic and therefore can be considered as a general linker in bioconjugation strategies for immunogenic applications.

After proving the good efficiency of the Ugi-conjugation with oxo-functionalized CPs of *S. pneumoniae*, we sought to address the bioconjugation scope with the antigen of *Salmonella enterica* serovar Typhi (*S.* Typhi), namely polysaccharide Vi (CPsVi). This is a very large biomolecule featuring a repetitive 2-acetamido-galacturonic acid unit.^28^ Typhoid fever is recognized as a world health problem with an incidence of 20 million cases per year,^29^ most of which are attributed to the Gram-negative bacterium *S.* Typhi.

**Scheme 2:**
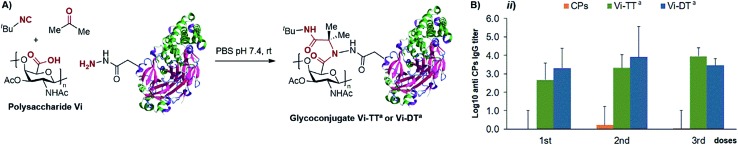
(A) Multicomponent conjugation of CPsVi to hydrazide-activated DT and TT. (B) Anti-Vi IgG titers elicited in mice by conjugates Vi-TT^a^ and Vi-DT^a^, as determined by ELISA.

As depicted in Scheme 2A, this polysaccharide was employed as the carboxylic acid component for the multicomponent conjugation to TT^a^ and DT^a^, using an excess of *t*-butyl isocyanide and acetone as the two other components. In each case, a solution of acetone/water/PBS (pH 7.4) was prepared and the protein was added to enable the initial formation of the imine intermediate, following addition of the CPsVi (carboxylic acid) and the isocyanide. The reactions were also monitored by SE-HPLC for a period of 8 h to 96 h, proving that after 72 h the amount of non-conjugated protein was less than 20%. Further purification by preparative SE-HPLC allowed complete removal of the non-conjugated protein (see the ESI†). Thus, the Vi-TT^a^ and Vi-DT^a^ glycoconjugates showed polysaccharide/protein ratios of 1 : 2.5 and 2 : 1, respectively, which are both in the range accepted by the WHO for vaccination. In the first case, a 1 : 2.5 ratio does not convey the presence of non-conjugated protein (removed by SE-HPLC), but that the cross-linked lattice matrix incorporating the two macromolecules simply has a higher protein content.^30^ A dot-blot experiment with a specific anti-Vi monoclonal antibody (mAb) was employed to assess the antigenicity of the resulting glycoconjugates, using the Vi-TT^a^ conjugate as an example (see the ESI†). This experiment proved the specific recognition of the Vi-TT^a^ conjugate by the anti-Vi mAb, thus confirming the preservation of the Vi-antigenic determinant upon bioconjugation. Both Vi-TT^a^ and Vi-DT^a^ conjugates – adsorbed on AlPO_4_ – were employed for the immunization in Balb/c mice and the IgG induced response was evaluated by ELISA. As shown in Scheme 2B, both glycoconjugates were able to induce a T-dependent immune response, proving the success of this type of multicomponent bioconjugation also with carboxylic acid-functionalized CPs.

After demonstrating the efficacy of the Ugi-conjugation process with both oxo- and carboxylic acid-functionalized CPs, we aimed to address a synthetic challenge not attainable for any other known bioconjugation method, *i.e.*, the conjugation of two CPs to a carrier protein in a single step. The main advantage of this type of multicomponent bioconjugation is that it allows for a more straightforward preparation of multivalent glycoconjugates, including the so-called multicomponent self-adjuvanting vaccines. This latter class of multivalent unimolecular constructs has attracted significant attention, especially in the field of anticancer vaccines including poorly immunogenic B-cell epitopes.^31^

The concept has been recently extended to antibacterial multivalent vaccines including either multiple CPs^32^ or a CP and an adjuvant^33^ conjugated to a carrier. Nevertheless, such multivalent glycoconjugates have been prepared through the incorporation of each component in separate conjugation steps.^32^,^33^ Herein we describe, for the first time, a multicomponent bioconjugation approach that enables the incorporation of two different polysaccharide antigens to a carrier protein in one pot, thus leading to a unique class of multivalent unimolecular glycoconjugate.

**Scheme 3:**
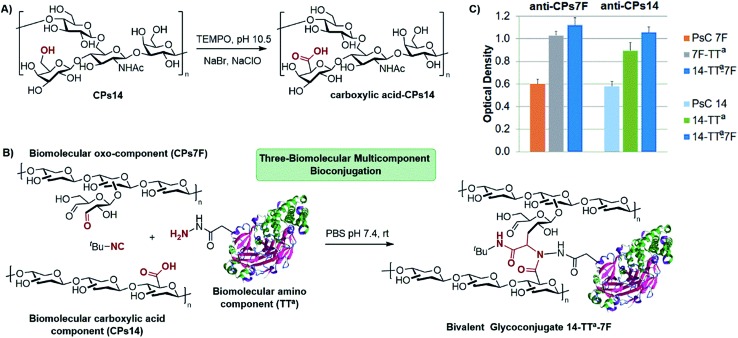
(A) Generation of carboxylic acid groups on CPs14 by TEMPO oxidation. (B) Multicomponent bioconjugation of carboxylic acid-functionalized CPs14 and oxo-functionalized CPs7F of *S. pneumoniae* to activated TT as the amino-component. (C) Antigenicity evaluation by ELISA of bivalent glycoconjugate 14-TT^a^-7F, in comparison with their individual CPs and monovalent conjugates using the specific anti-CPs14 and anti-CPs7F antibodies.

To implement this concept, we focused on the preparation of a glycoconjugate including two different CPs of *S. pneumoniae*. For this, CPs14 was subjected to a controlled oxidation with TEMPO to generate carboxylic acid functionalities at position 6 of multiple monosaccharide units (Scheme 3A, 1 carboxylic group every 2.8 repetitive units). As depicted in Scheme 3B, the oxo-functionalized CPs7F and hydrazide-activated TT were chosen as oxo and amino components, respectively, leading to the bivalent glycoconjugate CPs14-TT^a^-CPs7F. The conjugation procedure was conducted for 72 h using a 1.5 : 1 (w/w) ratio of each functionalized CP to the carrier protein and an excess of *t*-butyl isocyanide. The resulting glycoconjugate was purified by diafiltration and then analyzed by SE-HPLC to determine the amount of non-conjugated protein, *i.e.* 15%, which is acceptable for vaccination^27^ (see the ESI†). From the synthetic point of view, it is clear that the multicomponent reaction can only take place through the attachment of both functionalized polysaccharides to the carrier protein, which *per se* is a guarantee of the formation of the bivalent glycoconjugate. The polysaccharide/protein ratio in CPs14-TT^a^-CPs7F was 1 : 1.4, which is also in the range accepted for PCVs.^27^ The CPs14/CPs7F ratio turned out to be 1.2 : 1, as determined by quantitative NMR experiments (see the ESI†).

At this stage, it was important to assess whether the antigenicity on each individual CP was preserved at the bivalent construct. As depicted in Scheme 3C, the bivalent conjugate CPs14-TT^a^-CPs7F was analyzed by indirect ELISA with the anti-CPs14 and anti-CPs7F specific antibodies, using both the natural CPs and the monovalent conjugates CPs14-TT^a^ and CPs7F-TT^a^ as references. Hence, CPs14-TT^a^-CPs7F showed a marked specific recognition by both the anti-CPs14 and anti-CPs7F antibodies, in the same range as those of the monovalent conjugates. This analysis proved the dual antigenicity of the bivalent glycoconjugate and confirmed the integrity of the antigenic determinant of each CP after the multicomponent bioconjugation.

**Fig. 2:**
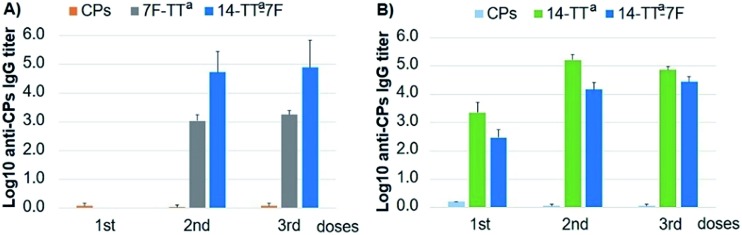
Assessment of the double immunogenicity of the glycoconjugate CPs14-TT^a^-CPs7F in comparison with the individual CPs and the monovalent conjugates, as determined by ELISA. IgG titers elicited in mice against (A) CPs7F and (B) CPs14.

A crucial issue for the scope of this strategy was to assess whether the bivalent glycoconjugate could be able to induce a simultaneous T-dependent response against both CPs14 and 7F. To evaluate such a double immunogenicity, we compared the IgG titers elicited in mice by the conjugate CPs14-TT^a^-CPs7F with those displayed by each monovalent glycoconjugate and the natural CPs. As shown in Fig. 2, the bivalent glycoconjugate showed an immunogenic behavior equivalent to those of its monovalent analogs, which is indeed a remarkable result. Thus, not only were the levels of IgG titers similar but also the type of response, with anti-7F antibodies being elicited after the second dose and the anti-14 ones after the first dose. These results prove the double immunogenic character of the bivalent glycoconjugate CPs14-TT^a^-CPs7F and the potential of this bioconjugation methodology for the development of multivalent vaccine candidates.

The functional antibacterial antibodies elicited by the monovalent conjugates CPs14-TT^a^ and CPs7F-TT^a^ as well as the bivalent CPs14-TT^a^-CPs7F were determined from opsonophagocytic assays (OPA) against target pneumococcal serotypes 14 and 7F. Opsonophagocytosis represents the main mechanism of host defense against pneumococcal disease, and although specific OPA antibody responses do not directly correlate with protection against infection, they are commonly accepted to correlate with vaccine-induced protection.^34^ OPA titers (pool of last immunization bleed-sera in 10 mice) were calculated, according to a described method,34*c* as the reciprocal of the sera of dilution that caused a 50% reduction (mediated killing) of the assay bacteria, compared to the control. The OPA titers shown in Table 1 confirm the functional character of the antibodies elicited by the three glycoconjugates, as a value higher than 8 (*i.e.*, dilution greater than 1 : 8) is required for a PCV to induce protection.^27^ These results suggest that immunization with the bivalent CPs14-TT^a^-CPs7F has the potential to induce a protective response for both serotypes 14 and 7F similar to that displayed by a combination of the individual monovalent glycoconjugates.

**Table 1:** Pneumococcal serotypes 14 and 7F OPA assays for the immune sera of conjugates CPs14-TT^a^, CPs7F-TT^a^ and CPs14-TT^a^-CPs7F

Conjugate	OPA titers against CPs14	OPA titers against CPs7F
CPs14-TT^a^	>256	—
CPs7F-TT^a^	—	128
CPs14-TT^a^-CPs7F	>256	128

## Conclusions

We have developed a new strategy for the preparation of antibacterial vaccine candidates using a multicomponent bioconjugation method. For the first time, properly functionalized CPs of pathogenic bacteria, such as *S. pneumoniae* and *S.* Typhi, were conjugated to tetanus and diphtheria toxoids by means of the Ugi reaction, leading to glycoconjugates with proven antigenic and immunogenic character. The multicomponent nature of the process allows for the simultaneous conjugation of two different CPs to a carrier protein in a single step, thus leading to a new type of multivalent unimolecular glycoconjugate. Both the good immunogenicity and the easy preparation of the bivalent glycoconjugate open many avenues of possibilities for the future development of conjugate vaccines of high valences, without the need for using a combination of so many conjugates. This strategy may also be amenable for the development of multicomponent, self-adjuvanting anticancer vaccines. Whereas various polyvalent antibacterial vaccines are readily available through co-formulation of their univalent glycoconjugates, some practical benefits of this multicomponent bioconjugation might be considered in future, for example, the lower number of conjugation/purification steps and, very importantly, the lower amount of carrier protein required to guarantee the T-dependent response of multiple carbohydrate antigens. This is especially promising for avoiding the phenomenon of epitopic suppression that often shows up when high carrier epitopic loads are employed in polyvalent or combination vaccine formulations.^35^ Our ongoing endeavor towards producing antibacterial vaccine candidates based on multivalent unimolecular glycoconjugates, including those with CPs of different bacteria, will be described in due course.

## Author contributions

D. G. R., Y. M. and J. C. conceived the idea of the work. Y. M., J. C., A. R. H. and A. Z. performed most of the experimental work. R. G., J. P. and A. V. V. carried out the analysis and characterization. D. G.-R., Y. V., D. S., J. C. and V. V.-B contributed to the bioconjugation strategy and the immunological evaluation. L. M. R. conducted the OPA assays. D. G. R. and Y. M. prepared the manuscript with contribution of all authors to the discussions.

## Conflicts of interest

There are no conflicts to declare.

## Supplementary Material

Supplementary information
