# Supplementary material for: Multicomponent polysaccharide–protein bioconjugation in the development of antibacterial glycoconjugate vaccine candidates
Source: Chem Sci. 2018 Jan 19;9(9):2581–8. doi: 10.1039/c7sc05467j (PMC5897956; doi:10.1039/c7sc05467j)
Supplement: Supplementary file 1 [file SC-009-C7SC05467J-s001.pdf]

Electronic Supplementary Material (ESI) for Chemical Science.
This journal is © The Royal Society of Chemistry 2018

Electronic Supplementary Information

Multicomponent Polysaccharide-Protein Bioconjugation in the Development of Antibacterial Glycoconjugate Vaccine Candidates

Yanira Méndez,[a,†] Janoi Chang,[b,†] Ana R. Humpierre,[a] Abel Zanuy,[b] Raine Garrido,[b] Aldrin V. Vasco,[a,c] Jessy Pedroso,[b] Darielys Santana,[b] Laura M. Rodríguez,[b] Dagmar García-Rivera,[b] Yury Valdés,[b] Vicente Vérez-Bencomo[b,]* and Daniel G. Rivera,[a,c]*

[a]Center for Natural Products Research, Faculty of Chemistry, University of Havana, Zapata y G, Havana 10400, Cuba

[b]Finlay Institute of Vaccines, Ave 27 Nr. 19805, Havana 10600, Cuba

[c]Department of Bioorganic Chemistry, Leibniz Institute of Plant Biochemistry, Weinberg 3, 06120, Halle/Saale, Germany.

[†]These authors contributed equally to this work.

Table of contents

Supporting figures, tables and schemes 4

Table S1: Structure of the repeating units of CPs employed in this work 4

Figure S1. SE-HPLC traces of natural, fragmented and oxidized (ox) CPs (TSK 5000 PW column)....................4

Figure S2. ELISA analysis of the antigenicity of oxCPs 5

Figure S3. SE-HPLC traces of multicomponent conjugation of CPs14 monitoring (Superose® 12 column)....................6

Figure S4. SDS-PAGE analysis of Ugi-derived glycoconjugates compared with the free proteins 6

Figure S5. ^{1}H-NMR-based determination of the CPs14 repeating unit identity at 27 °C (400 MHz, D_2O,) 7

Figure S8. ^{1}H-NMR-based determination of CPsVi repeating unit identity at 52 °C (400 MHz, D_2O) 8

Figure S9. Quantitative ^{1}H-NMR-based determination of identity and content of CPs7F and CPs14 at 25 °C (500 MHz, D_2O) in Ugi-bivalent conjugate CPs14-TT^a-CPs7F. 9

Figure S10. Analysis of the stability of the conjugate CPs14-TT^a up to 3 months after the conjugation (Superose® 12). 10

Figure S11. Analysis of the stability of the conjugate CPs14-TT^a up to 3 months after the conjugation (TSK 5000) 11

Table S2. Free protein percentages and carbohydrate-protein ratio of conjugates CPs14-TT[a], CPs14-DT[a], CPs7F-TT[a], CPs7F-DT[a], CPs9V-TT[a], Vi-TT[a] and Vi-DT[a] 12

Methods and Results 12

General information 12

Synthetic procedure for the fragmentation and periodic oxidation of CPs7F from *Streptococcus pneumoniae* 13

Synthetic procedure for the fragmentation and periodic oxidation of CPs9V from *Streptococcus pneumoniae* 13

Synthetic procedure for the fragmentation and periodic oxidation of CPs14 from *Streptococcus pneumoniae* 14

Synthetic procedure for the oxidation of CPs14 from *Streptococcus pneumoniae* with TEMPO 14

General synthetic procedure for the modification of proteins with hydrazine 14

General synthetic procedure for the conjugation of carbonyl containing CPs by Ugi-4CR 15

General synthetic procedure for the conjugation of CPsVi from *Salmonella enterica* serovar Typhi by Ugi-4CR 16

Synthesis of the conjugate CPs14-non-activated BSA (CPs14-BSA) 16

Synthesis of the conjugate CPs14-activated BSA (CPs14-BSA[a]) 17

Synthesis of the conjugate CPs 14- non-activated DT (CPs14-DT) 17

Synthesis of the conjugate CPs 14- non-activated TT (CPs14-TT) 17

Synthesis of the conjugate CPs 14- activated TT (CPs14-TT[a]) 17

Synthesis of the conjugate CPs 14- activated DT (CPs14-DT[a]) 18

Synthesis of the conjugate CPs7F-TT[a] 18

Synthesis of the conjugate CPs7F-DT[a] 19

Synthesis of the conjugate CPs9V-TT[a] 20

Synthesis of the conjugate dextran-BSA[a] 20

Synthesis of the conjugate CPsVi-TT[a] 21

Synthesis of the conjugate CPsVi-DT[a] 22

Synthesis of the conjugate CPs14-TT[a]-CPs7F 23

Colorimetric determinations 24

Sulfuric acid-orcinol method 24

Biphenyl method 24

Lowry's method 24

Hestrin's method 25

Electrophoresis on polyacrylamide gel (SDS-PAGE) 25

Immunization schemes 26

ELISA analysis 27

Determination of the antibodies (IgG) toward the CPs 27

Determination of the antigenicity of fragmented CPs and OxCPs 28
Determination of the antibodies elicited by the Ugi-linkage 29
Determination of the antigenicity of the conjugate CPsVi-TT[a] by dot-blot 29
Opsonophagocytic assays (OPA)..30

Supporting figures, tables and schemes

Table S1: Structure of the repeating units of CPs employed in this work

Polysaccharide	Repeating unit
Streptococcus pneumoniae	
CPs7F[2]	→6)-[β-D-Gal*p*-(1→2)]-α-D-Gal*p*-(1→3)-β-L-Rha*p*OAc-(1→4)-β-D-Glc*p*-(1→3)-[α-D-Glc*p*NAc-(1→2)-α-L-Rha*p*--(1→4)]-β-D-Gal*p*NAc-(1→
Cps9V[3]	→4)-α-D-Glc*p*A-(1→3)-α-D-Gal*p*-(1→3)-β-D-Man*p*NAc-(1→4)-β-D-Glc*p*-(1→4)-α-D-Glc*p*-(1→ (+Ac)
CPs14[1]	→4)-β-D-Glc*p*-(1→6)-[β-D-Gal*p*-(1→4)]-β-D-Glc*p*NAc-(1→3)-β-D-Gal*p*-(1→
***Salmonella enterica* Typhi Vi[1]**	→)-α-D-Gal*p*NAcA(3OAc)-(1→

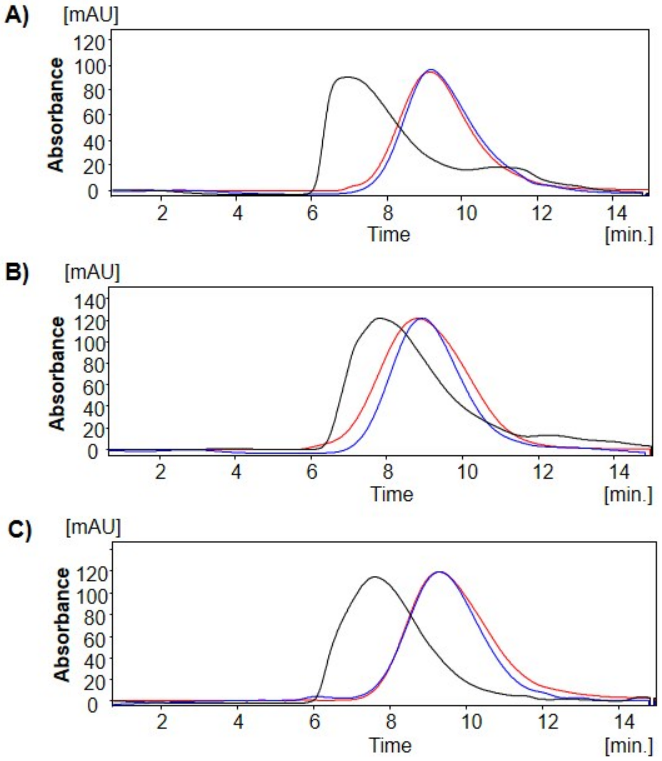

Figure S1. SE-HPLC traces of natural, fragmented and oxidized (ox) CPs (TSK 5000 PW column). The samples were analyzed at 1.0 ml/min of flow in NaCl 0.9 %, with an IR detector. (A) Overlapped traces of natural CPs7F (black line), fragmented CPs7F (red line) and oxCPs7F (blue line). (B) Overlapped traces of natural CPs9V (black line), fragmented CPs 9V (red line) and oxCPs 9V (blue line). (C) Overlapped traces of natural CPs14 (black line), fragmented CPs14 (red line) and oxCPs14 (blue line).

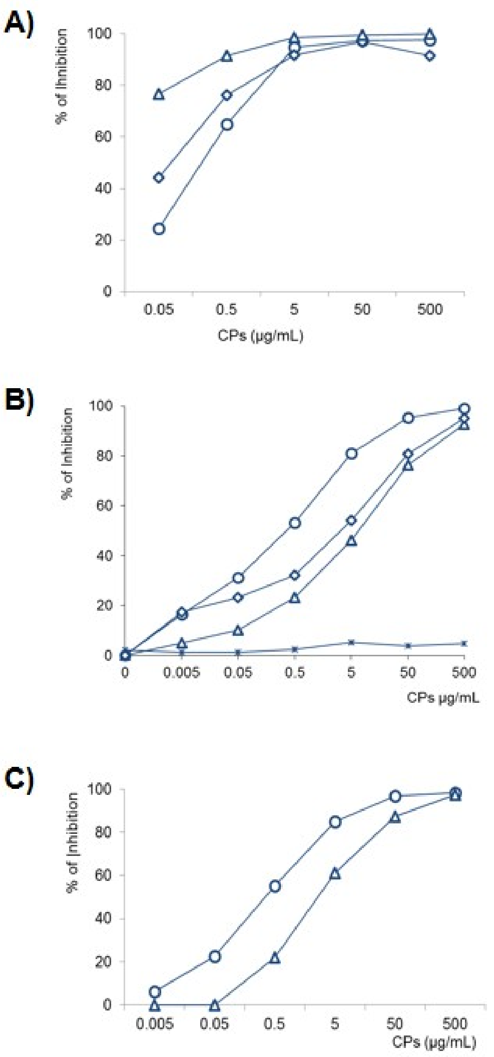

Figure S2. ELISA analysis of the antigenicity of oxCPs. Inhibition curve of the specific rabbit serum (from Statens Serum Institute, Copenhagen) against A) oxCPs14; B) oxCPs7F and C) oxCPs9V. ELISAs were performed on a plate coated with 10µg/mL of the CPs. Dilutions of 1:20000 were used for the three specific serum and a dilution of 1:10000 was used for the anti-IgG-HRP conjugate. Natural CPs (O), fragmented CPs (◊), oxCPs (Δ) and no related CPs (*) in the three graphics.

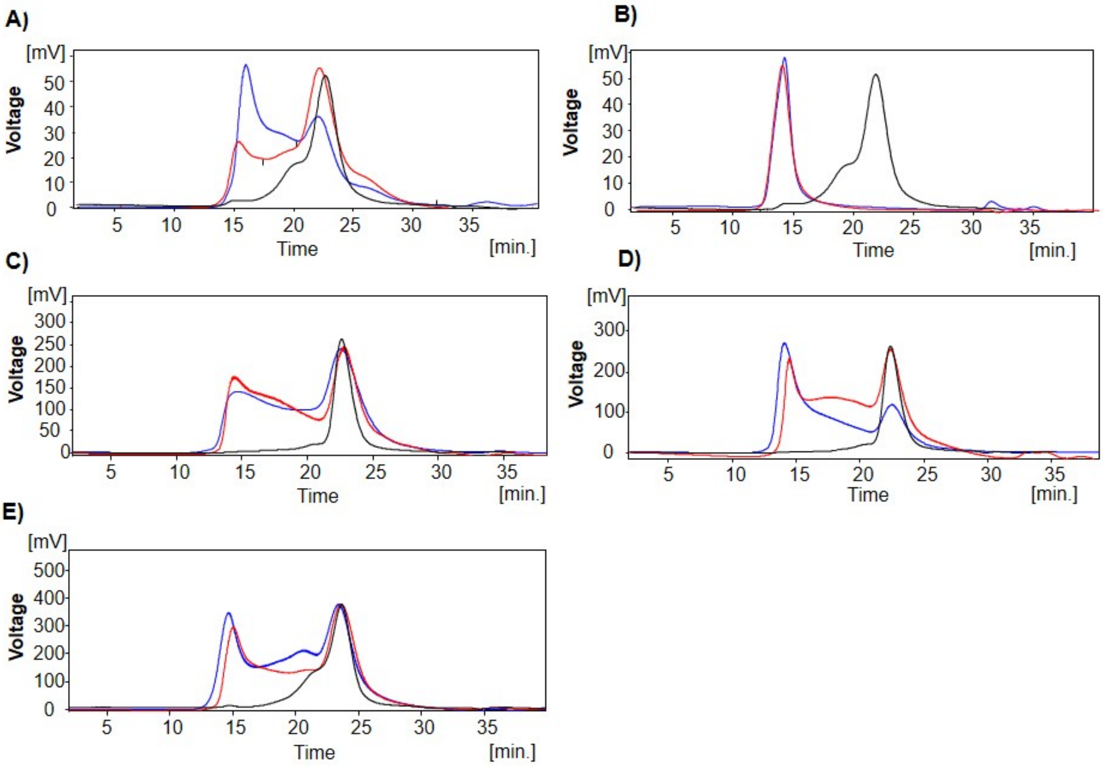

Figure S3. SE-HPLC traces of multicomponent conjugation of CPs14 monitoring (Superose® 12 column). HPLC traces of the conjugation of oxCPs14 to A) non-activated TT, B) hydrazide-activated TT C) non-activated BSA, D) hydrazide-activated BSA and E) non-activated DT. The samples were analyzed at a flow of 0.5 ml/min in PBS, pH=7.0, with a UV detector (λ = 206 nm). All panels show the reaction conversion from BSA, DT or TT before reaction (black line), after 4 hours (red line) and after 48 hours (blue line).

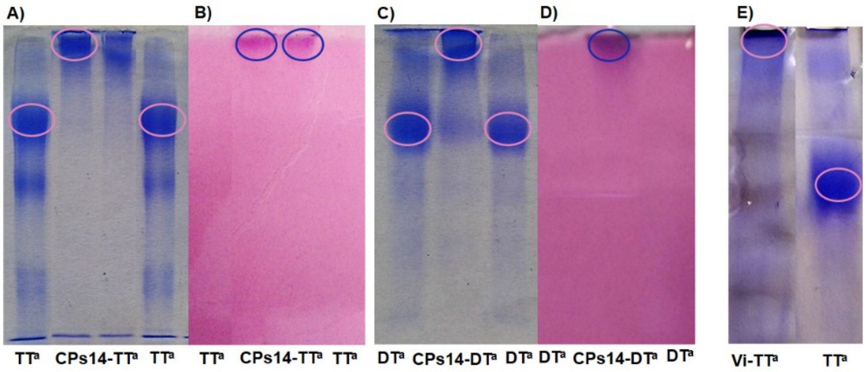

Figure S4. SDS-PAGE analysis of Ugi-derived glycoconjugates compared with the free proteins. A) TT^a and conjugate CPs14-TT^a with Coomasie staining; B) TT^a and conjugate CPs14-TT^a with Fuchsin staining; C) DT^a and conjugate CPs14-DT^a with Coomasie staining; D) DT^a and conjugate CPs14-DT^a with Fuchsin staining; E) TT^a and conjugate Vi-TT^a with Coomasie staining**.** The experiments were run at 40mM, constant intensity and free voltage; with a concentrating gel with 10 % of polyacrylamide for DT and 7 % of polyacrylamide for TT, and resolving gel with 4 % of polyacrylamide.

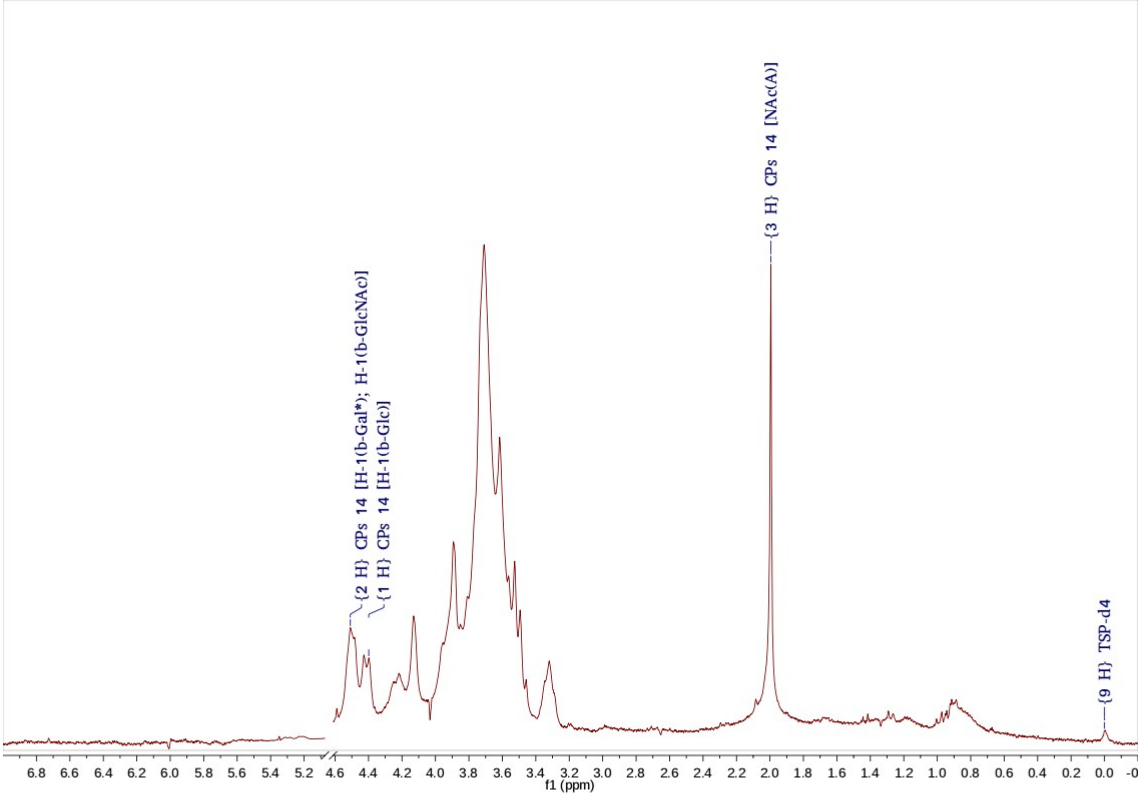

Figure S5. ^{1}H-NMR-based determination of the CPs14 repeating unit identity at 27 ^{o}C (400 MHz, D_2O,) in Ugi-conjugate CPs14-TT. Note: b-Gal* -[β-D-Gal*p*-(1→4)]-

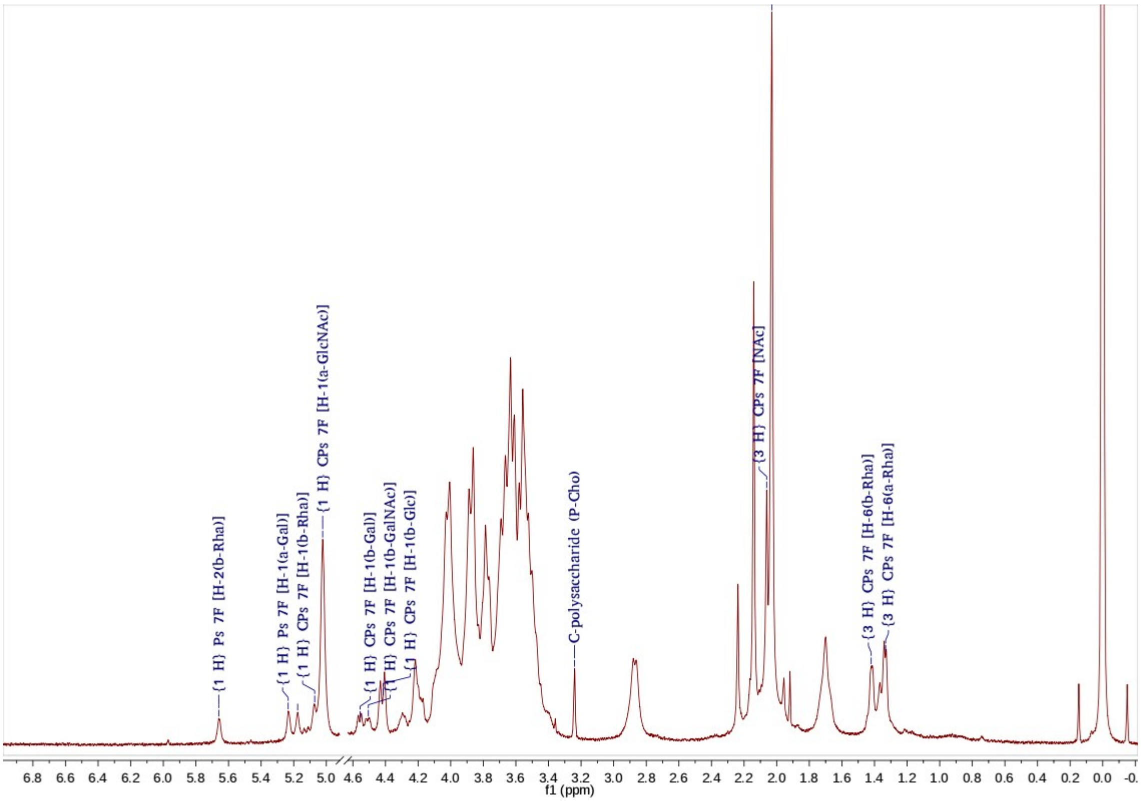

Figure S6. ^{1}H-NMR-based determination of the CPs7F repeating unit identity at 25 ^{o}C (400 MHz, D_2O) in Ugi-conjugate CPs7F-TT[a].

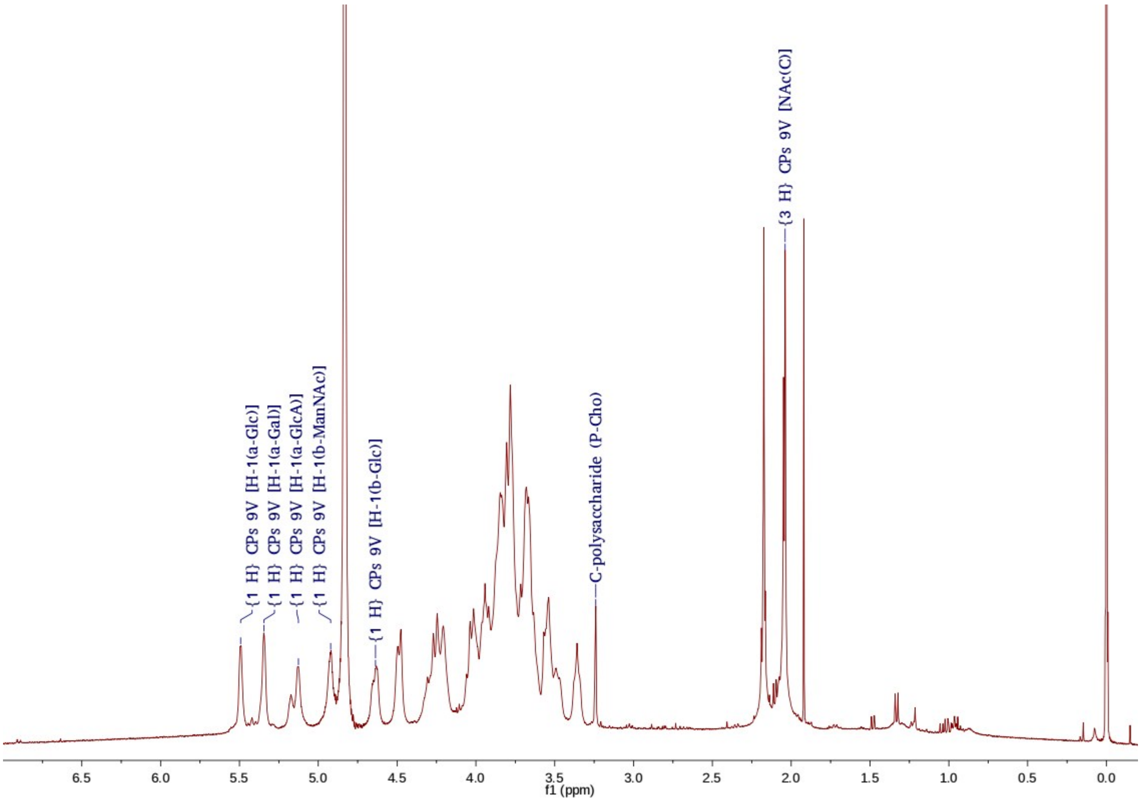

Figure S7. ^{1}H-NMR-based determination of the CPs9V repeating unit identity at 25 ^{o}C (400 MHz, D_2O) in Ugi-conjugate CPs9V-TT[a].

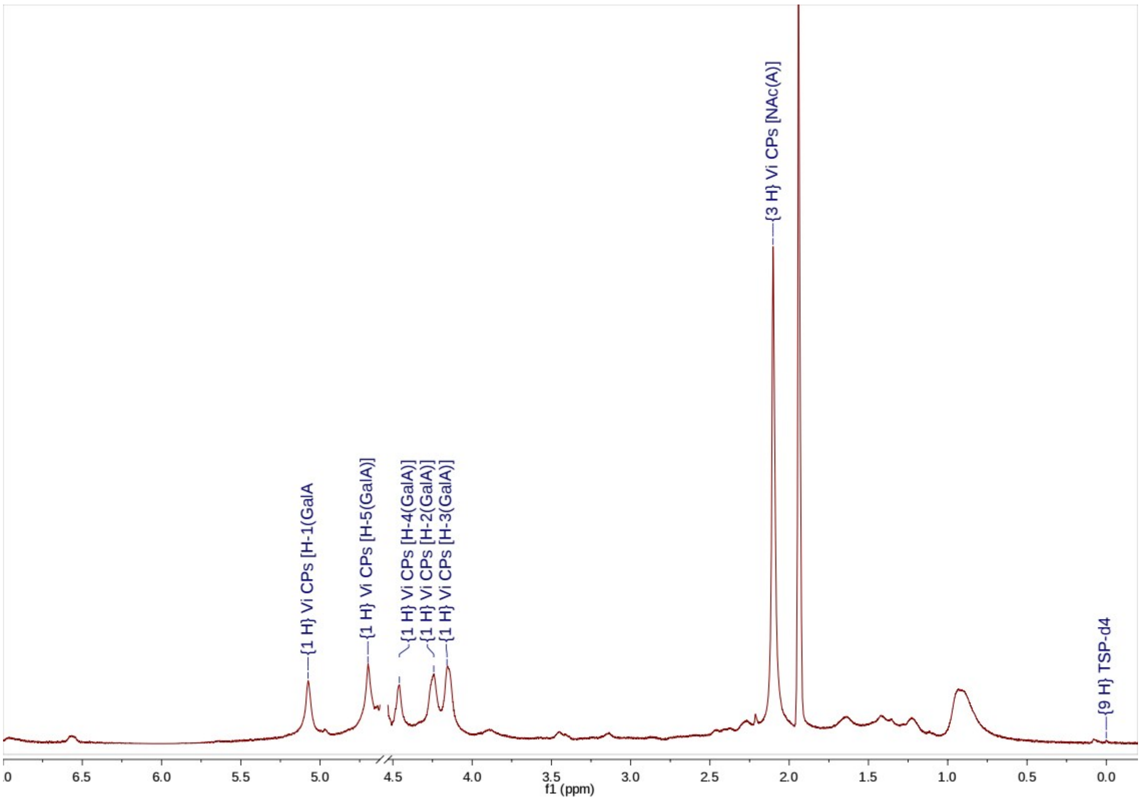

Figure S8. ^{1}H-NMR-based determination of CPsVi repeating unit identity at 52 ^{o}C (400 MHz, D_2O) in Ugi-conjugate Vi-DT[a].

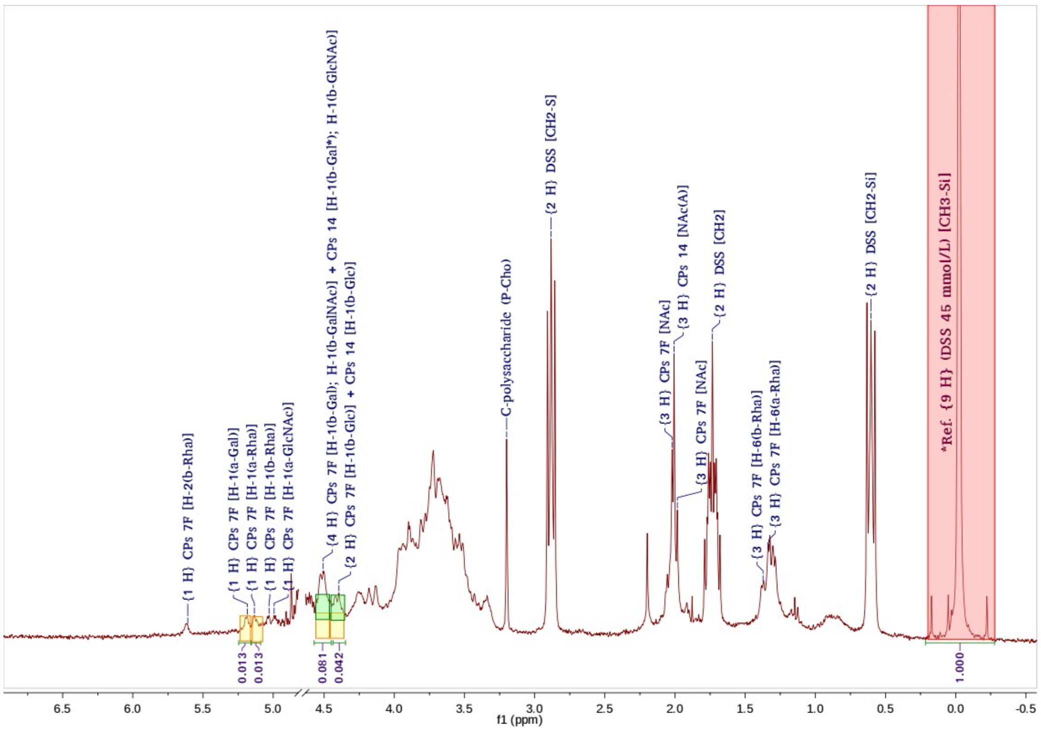

Figure S9. Quantitative ^{1}H-NMR-based determination of identity and content of CPs7F and CPs14 at 25 ^{o}C (500 MHz, D_2O) in Ugi-bivalent conjugate CPs14-TT^a-CPs7F.

Proton qNMR

The proton qNMR experiment was set with a recycled time of 30 s, pulse duration P1 adjusted at 45°and 32 scans. To obtain the spectra, the Free Induction Decay (FID) was processed with a Fourier Transform apodizated by an exponential function (LB 0.3 Hz). To guarantee the signal integration process baseline adjustment was required. The spectral data were acquired and transformed under TopSpin 1.3 software pack.

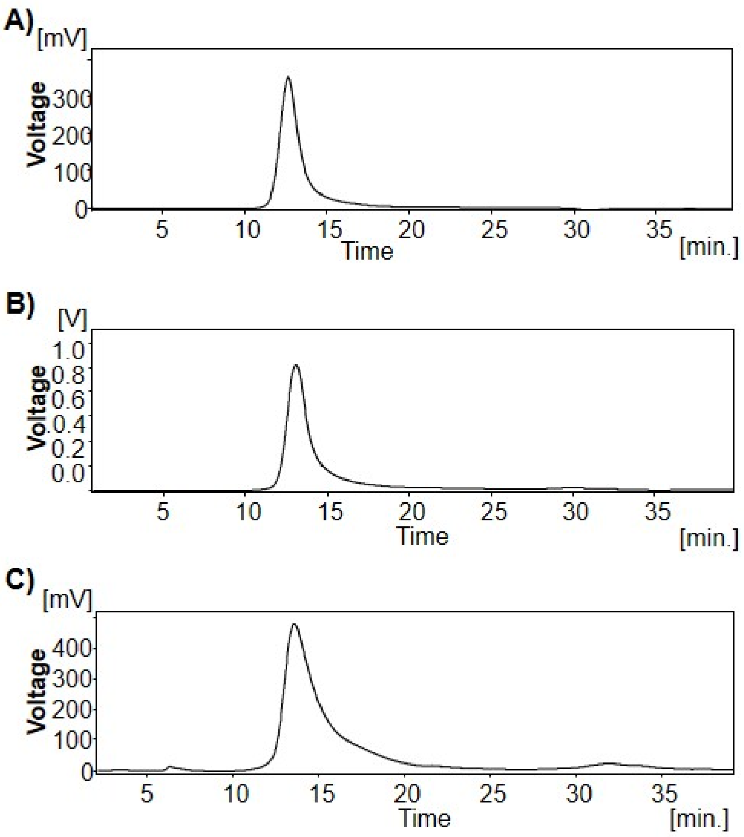

Figure S10. Analysis of the stability of the conjugate CPs14-TT[a] up to 3 months after the conjugation (Superose® 12). A) SE-HPLC traces (Superose® 12) after the purification of the conjugate. B) SE-HPLC traces (Superose® 12) after 45 days. C) SE-HPLC (Superose® 12) after 3 months.

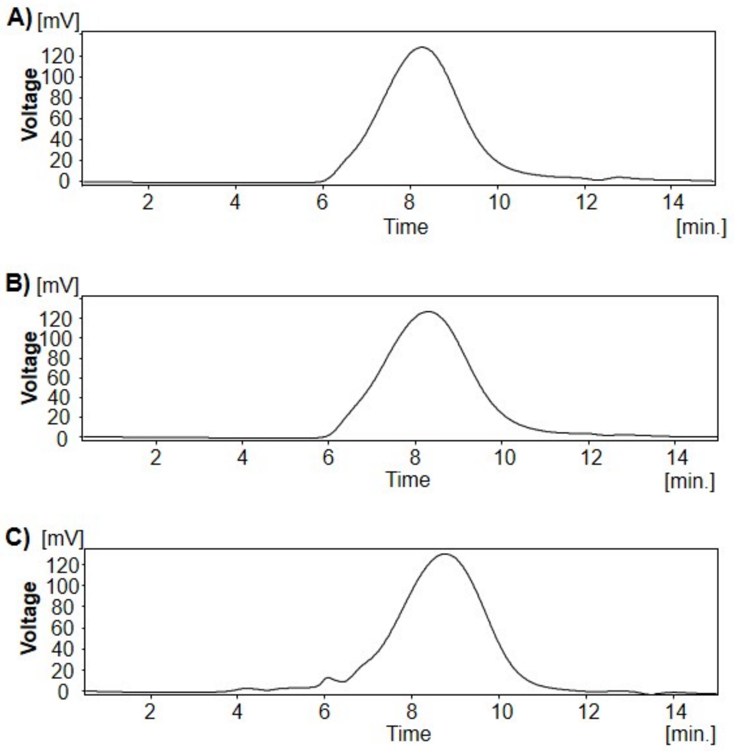

Figure S11. Analysis of the stability of the conjugate CPs14-TT[a] up to 3 months after the conjugation (TSK 5000). A) SE-HPLC traces (TSK 5000; Kd = 0.277) after the purification of the conjugate. B) SE-HPLC traces (TSK 5000; Kd = 0.264) after 45 days. C) SE-HPLC traces (TSK 5000; Kd = 0.252) after 3 months.

Table S2. Free protein percentages and carbohydrate-protein ratio of conjugates CPs14-TT[a], CPs14-DT[a], CPs7F-TT[a], CPs7F-DT[a], CPs9V-TT[a], Vi-TT[a] and Vi-DT[a]

Conjugate	FPP %[a]	Conc. Protein[b] (mg/mL)	Conc. CPs[c, d] (mg/mL)	CPs/Protein
CPs14-TT[a]	0	1.97	0.90	1.0 : 2.2
CPs14-DT[a]	12.5	2.50	1.76	1.0 : 1.4
CPs7F-TT[a]	2.8	2.49	1.46	1.0 : 1.7
CPs7F-DT[a]	10	1.42	1.67	1.2 : 1.0
CPs9V-TT[a]	14	1.32	1.80	1.4 : 1.0
Vi-TT[a] 1st	0	0.46	1.74	1.0 : 3.8
Vi-TT[a] 2nd	0	1.21	3.03	1.0 : 2.5
Vi-DT[a]	0	0.30	0.61	2.0 : 1.0
CPs14-TT[a]-CPs7F	15	1.43	(CPs14/CPs7F) 1.2/1.0 [(d)]	1.0 : 1.4

[a]Estimated by HPLC in a Superose® 12 column , with manual integration of the area below the curve at the protein retention time
[b]Protein quantification using Lowry´s method[4]
[c]Carbohydrate quantification using the orcinol-sulfuric acid method for CPs14 and CPs7F conjugates,[5] the biphenyl method for CPs9V conjugates[6] and the Hestrin´s method[7] for CPsVi conjugates.
[d]Carbohydrate quantification using quantitative NMR.

Experimental Methods

General information

Proton nuclear magnetic resonance spectra were recorded on a Bruker/Avance DRX 500 (500 MHz) and on a Varian Mercury 400 (400 MHz) at 27 °C/52 °C and 25 °C, respectively. Samples of CPs as well as their conjugates were prepared by dissolving 10 mg in 0.6 mL of deuterated water (D_2O, Merck > 99.8 %), lyophilizing and dissolving them again three times. All chemical shifts are reported in ppm and were determined by using 3-(trimethylsilyl)-2,2,3,3-tetradeuteropropionic acid sodium salt (TSP-d_4) as internal standard. For proton qNMR was used 4,4-dimethyl-4-silapentanesodium sulfonate (DSS) 45 mmol/L as internal reference. Coupling constants (J) are reported in hertz (Hz). In most of all spectra it is possible to observe a signal at 3.22 ppm, belonging to the C-polysacharide, a common impurity in the CPs isolation process.

All the chromatography-destined solvents were distilled, dried and degasified following the reported procedures. All the reactants employed in the synthesis, characterization

and chromatography (including SE-HPLC and SDS-PAGE) were purchased from Merck, Fluka and Panreac.

Carbohydrate and protein contents were determined by using a spectrophotometer Jenway 6705 UV/Vis. A sulfuric acid-orcinol method[5] was employed for the carbohydrate determination in CPs14- and CPs7F- containing conjugates, a biphenyl based method[6] was employed for CPs9V- containing conjugates and the Hestrin´s method[7] was employed for CPsVi- containing conjugates. In all cases, the Lowry´s method[4] was employed for the protein quantification.

Synthetic procedure for the fragmentation and periodic oxidation of CPs7F from *Streptococcus pneumoniae*

Trifluoracetic acid (10 mM) was added to an aqueous solution of natural CPs7F (2mg/mL) and the reaction mixture was stirred at 70 ^{o}C for 2 h and 30 min. The resulting fragmented polysaccharide was diafiltrated through a series of regenerated cellulose membranes (10-100 kDa) with 10 water volumes each time, and 2-4 bar nitrogen pressure to afford CPs7F (10-100 kDa, 73 %). Fragmented CPs7F (5 mg/mL) was mixed with $NaIO_4$ (2 mM) and the reaction mixture was protected from light and stirred at 10 ^{o}C for 3 h. An excess of ethylene glycol was added to quench the reaction and the fragmented oxidized CPs7F was purified by diafiltration through a regenerated cellulose membrane (10 kDa) with 10 water volumes and 4 bar nitrogen pressure to afford oxCPs7F (1 C=O every 7 repetitive units (RU), 80 %).

Synthetic procedure for the fragmentation and periodic oxidation of CPs9V from *Streptococcus pneumoniae*

Trifluoracetic acid (50 mM) was added to an aqueous solution of natural CPs9V (2mg/mL) and the reaction mixture was stirred at 70 ^{o}C for 4 h. The resulting fragmented polysaccharide was diafiltrated through a series of regenerated cellulose membranes (10-100 kDa) with 10 water volumes each time, and 2-4 bar nitrogen pressure to afford CPs9V (10-100 kDa, 90 %). Fragmented CPs9V (5 mg/mL) was mixed with $NaIO_4$ (2 mM) and the reaction mixture was protected from light and stirred at room temperature for 2 h. An excess of ethylene glycol was added to quench the reaction and the fragmented oxidized CPs9V was purified by diafiltration through a regenerated cellulose membrane (10 kDa) with 10 water volumes and 4 bar nitrogen pressure to afford oxCPs9V (1 C=O every 7 RU, 79 %).

Synthetic procedure for the fragmentation and periodic oxidation of CPs14 from *Streptococcus pneumoniae*

Trifluoracetic acid (25mM) was added to an aqueous solution of natural CPs14 (2mg/mL) and the reaction mixture was stirred at 70 ^{o}C for 2h and 30 min. The resulting fragmented polysaccharide was diafiltrated through a series of regenerated cellulose membranes (10-100 kDa) with 10 water volumes each time, and 2-4 bar nitrogen pressure to afford CPs14 (10-100 kDa, 88.2 %). Fragmented CPs14 (5 mg/mL) was mixed with $NaIO_4$ (2 mM) and the reaction mixture was protected from light and stirred at room temperature for 3 h. An excess of ethylene glycol was added to quench the reaction and the fragmented oxidized CPs14 was purified by diafiltration through a regenerated cellulose membrane (10 kDa) with 10 water volumes and 4 bar nitrogen pressure to afford oxCPs14 (1 C=O every 7 RU, 92.3 %).

Synthetic procedure for the oxidation of CPs14 from *Streptococcus pneumoniae* with TEMPO

To a solution of fragmented CPs14 (10-100 kDa, (10 mg, 14.5 mmol relative to the repetitive unit mass)) in 20 mL of a buffer CO_3^{2-}/HCO_3^{-} (2M, pH = 10.5), TEMPO (0.042 eq., 0.533 mmol), NaBr (0.28 eq., 8 mmol) and NaClO (11 %) were added. After 4 h, the reaction was quenched with ethanol (10 eq.). The TEMPO-oxidized CPs14 was purified by diafiltration through a regenerated cellulose membrane (1 kDa) with 10 water volumes and 4 bar nitrogen pressure (oxidation degree: 1 -COOH every 2.8 RU, 78.5%).

General synthetic procedure for the modification of proteins with hydrazine

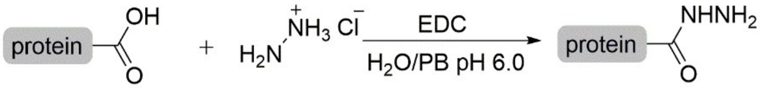

Scheme S1. Synthetic procedure for the activation of carrier proteins with hydrazine.

To a solution containing the detoxified protein with a concentration in the range from 3 to 5 mg/mL (determined by Lowry's method) in phosphate buffer (pH = 6.0), hydrazine hydrochloride and *N*-(3-dimethylaminopropyl)-*N'*-ethylcarbodiimide hydrochloride (EDC) 20mM are added. The reaction mixture is stirred for 4 hours at room temperature in the darkness. When the reaction is completed, the pH is adjusted to a value between 10.4 and 10.6. The product is purified by diafiltration through a regenerated cellulose membrane (30 kDa) with 10 volumes of a carbonate buffer (pH = 10.45), and 4 bar

nitrogen pressure. The activated protein is characterized by HPLC and colorimetric assays (TNBS and Lowry).

Tetanus toxoid (TT) and diphtheria toxoid (DT) reacted following the general synthetic procedure for the modification of proteins with hydrazine to afford activated TT and DT with the incorporation of 65-68 and 37-40 hydrazide groups per mol of toxoid, respectively. Bovine serum albumin (BSA) reacted following general synthetic procedure for the modification of proteins with hydrazine to afford hydrazide-activated BSA (39.4 hydrazide groups per mol of BSA, yield 80.5 %).

General synthetic procedure for the conjugation of carbonyl containing CPs by Ugi-4CR

To a solution containing the detoxified/non-activated protein (Scheme S2A) or the detoxified/hydrazide-activated protein (Scheme.S2B) in a saline phosphate buffer (pH = 7.4), the fragmented and periodate-oxidized polysaccharide is added in a 1:1 ratio related to the mass of the protein, together with a solution of acetic acid (10 %). The reaction mixture is stirred for 15 minutes at room temperature and then *tert*-butyl isocyanide is added. The reaction mixture is stirred for a specific period of time (depending on the protein and results of SE-HPLC monitoring). The conjugate is purified by diafiltration through a regenerated cellulose membrane (100 kDa) with 10 water volumes, and 2 bar of nitrogen pressure.

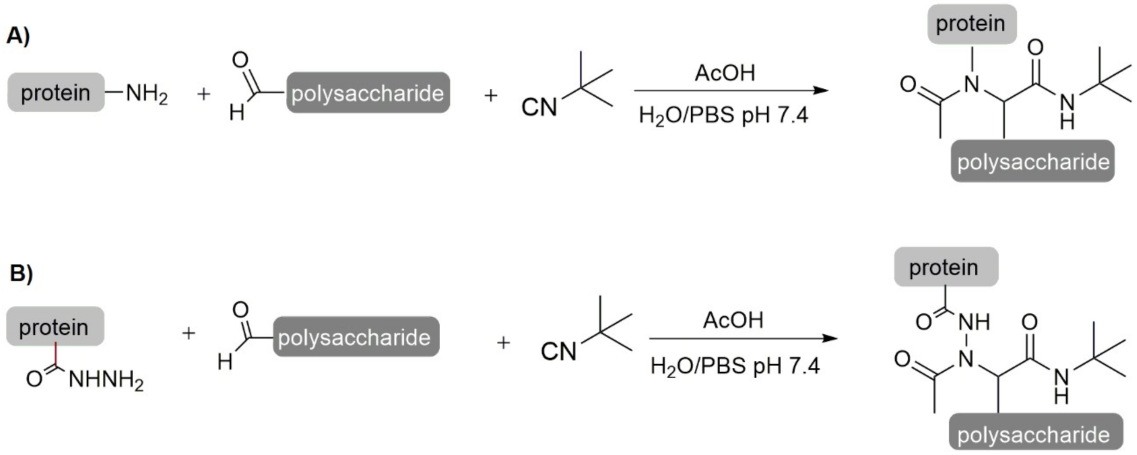

Scheme S2. Multicomponent conjugation of carbonyl containing CPs A) to a non-activated protein and B) to an activated protein.

General synthetic procedure for the conjugation of CPsVi from *Salmonella enterica* serovar Typhi by Ugi-4CR

To a solution containing the detoxified/non-activated protein (Scheme S3) or the detoxified/hydrazide-activated protein and acetone (excess) in a saline phosphate buffer (pH = 7.4), the natural polysaccharide is added in a 1:1 ratio related to the mass of the protein. The reaction mixture is stirred for 15 minutes at room temperature and then *tert*-butyl isocyanide is added. The reaction mixture is stirred for a specific period of time (depending on the protein and the results of SE-HPLC monitoring). The conjugate is purified by diafiltration through a membrane of regenerated cellulose (100 kDa) with 10 water volumes, and 2 bar nitrogen pressure, followed by preparative SE-HPLC Superose® 6 (diameter= 1.6 cm; longitude = 70 cm; sample volume = 4 mL (4 % of the total volume of the column), flux = 0.4 mL/min, UV detector at 200 nm).

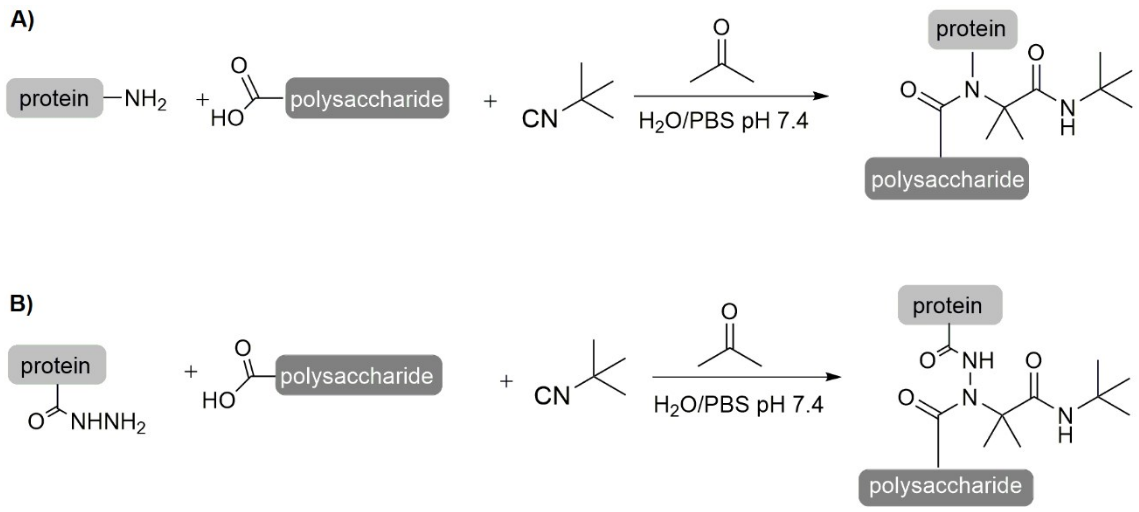

Scheme S3. Multicomponent conjugation of CPsVi from *Salmonella enterica* serovar Typhi A) to a non-activated protein and B) to an activated protein.

All reactions were monitored by SE-HPLC, with the employment of a Merck HPLC equipped with a Pharmacia LKB Pump P-500, and Superose® 12/ TSK 5000 as molecular exclusion gels. Analytical characterization of all purified glycoconjugates was carried out using the same method. The chromatographic data was analyzed using the software Clarity Chrom.

Synthesis of the conjugate CPs14-non-activated BSA (CPs14-BSA)

Native bovine serum albumin (BSA) (10 mg, 0.00015 mmol), fragmented and oxidized CPs14 from *Streptococcus pneumoniae* (100-10 kDa; 1 C=O every 7 RU (10 mg; 0.001 mmol)), AcOH at 10% (2 µL, 0.003 mmol) and *tb*NC (2 µL, 0.03 mmol) reacted in PBS

pH = 7.0 following the general procedure described above for the Ugi-conjugation of carbonyl containing CPs. The reaction was monitored by SE-HPLC (Superose® 12) after 4h and 48h in order to determine the conversion in this period of time (**Figure S3 C)**).

Synthesis of the conjugate CPs14-activated BSA (CPs14-BSA^a)

Hydrazide-activated bovine serum albumin (BSA^a) (10 mg, 0.00015 mmol), fragmented and oxidized CPs14 from *Streptococcus pneumoniae* (100-10 kDa; 1 C=O every 7 RU (10 mg; 0.001 mmol)), AcOH at 10% (2 µL, 0.003 mmol) and *tb*NC (2 µL, 0.03 mmol) reacted in PBS pH = 7.0 following the general procedure described above for the Ugi-conjugation of carbonyl containing CPs. The reaction was monitored by SE-HPLC (Superose® 12) after 4h and 48h in order to determine the conversion in this period of time (**Figure S3 D)**).

Synthesis of the conjugate CPs 14- non-activated DT (CPs14-DT)

Detoxified/ non-activated diphtheria toxoid (DT) (5 mg, 0.00008 mmol), fragmented and oxidized CPs14 from *Streptococcus pneumoniae* (100-10 kDa; 1 C=O every 7 RU (5 mg; 0.0005 mmol)), AcOH at 10% (2 µL, 0.003 mmol) and *tb*NC (2 µL, 0.03 mmol) reacted in PBS pH = 7.0 following the general procedure described above for the Ugi-conjugation of carbonyl containing CPs. The reaction was monitored by SE-HPLC (Superose® 12) after 4h and 48h in order to determine the conversion in this period of time (**Figure S3 E).**

Synthesis of the conjugate CPs 14- non-activated TT (CPs14-TT)

Detoxified/ non-activated tetanus toxoid (TT) (5 mg, 0.00003 mmol), fragmented and oxidized CPs14 from *Streptococcus pneumoniae* (100-10 kDa; 1 C=O every 7 RU (10 mg; 0.001 mmol)), AcOH at 10% (2 µL, 0.003 mmol) and *tb*NC (2 µL, 0.03 mmol) reacted in PBS pH = 7.4 following the general procedure described above for the Ugi-conjugation of carbonyl containing CPs. The reaction was monitored by SE-HPLC (Superose® 12) after 4h and 48h in order to determine the conversion in this period of time (**Figure S3 A**).

Synthesis of the conjugate CPs 14- activated TT (CPs14-TT^a)

Detoxified/ hydrazide-activated TT (TT^a) (5 mg, 0.00003 mmol), fragmented and oxidized CPs14 from *Streptococcus pneumoniae* (100-10 kDa; 1 C=O every 7 RU (7 mg, 0.0007 mmol)), AcOH at 10% (2 µL, 0.003 mmol) and *tb*NC (2 µL, 0.03 mmol)

reacted in PBS pH = 7.4 following the general procedure described above for the Ugi-conjugation of carbonyl containing CPs. The reaction was monitored by SE-HPLC (Superose® 12). The purified conjugate was also characterized by SE-HPLC (Superose® 12: conjugate retention time (rt.) = 12.86 min) 100 %), SDS-PAGE (**Figure S4 A) and B)**) and **^{1}H-NMR (400 MHz, D_2O):** δ (ppm) = 4.55 (anomeric C*Hs*, →4)-β-D-Glc-(1→ /D-Gal-(1→); 4.44 (anomeric C*H,* →6)-β-[→4)]-β-D-GlcNAc-(1→); 2.04 (CH_3, N*H*Ac, →4)-β-D-GlcNAc-(1→) (**Figure S5**)

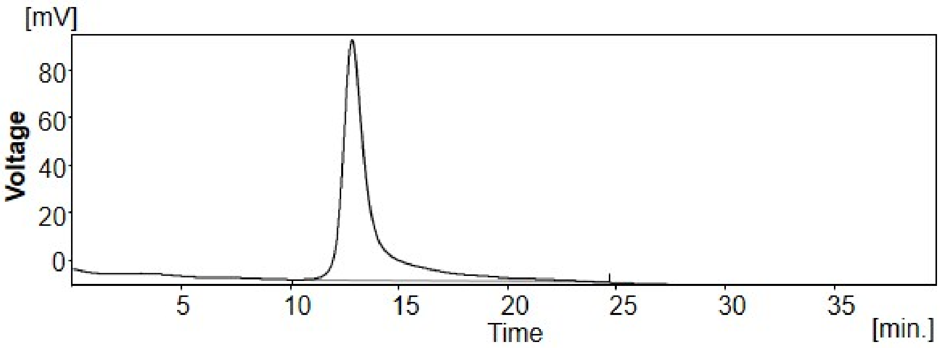

Figure S12. SE-HPLC traces (Superose® 12) of purified conjugate CPs14-TT[a]

Synthesis of the conjugate CPs 14-activated DT (CPs14-DT[a])

Detoxified/hydrazide-activated DT (DT[a]) (5 mg, 0.00008 mmol), fragmented and oxidized CPs14 from *Streptococcus pneumoniae* (100-10 kDa; 1 C=O every 7 RU (10 mg; 0,001 mmol), AcOH at 10% (2 µL; 0.003 mmol) and *tb*NC (2 µL; 0.03 mmol) reacted in PBS pH = 7.4 following the general procedure described above for the Ugi-conjugation of carbonyl containing CPs. The purified conjugate was characterized by SE-HPLC (Superose® 12: conjugate rt. = 14.37 min, 87.5 %; non-conjugated protein rt. = 23.95 min, 12.5 %) and SDS-PAGE (**Figure S4 C and D).**

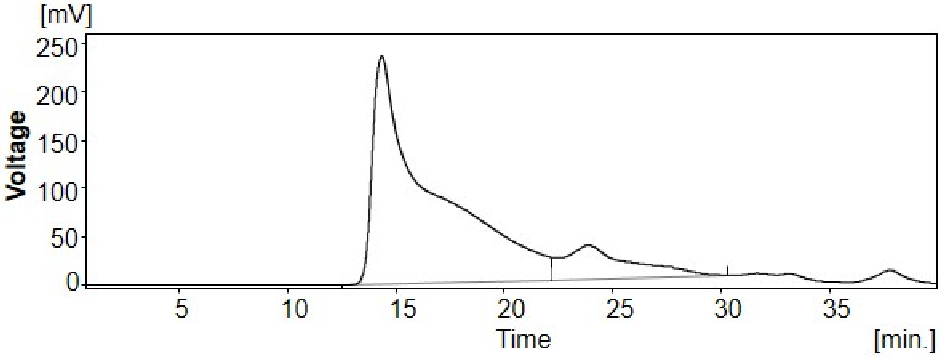

Figure S13. SE-HPLC traces (Superose® 12) of purified conjugate CPs14-DT[a]

Synthesis of the conjugate CPs7F-TT[a]

TT[a] (7 mg, 0.00005 mmol), fragmented and oxidized CPs7F from *Streptococcus pneumoniae* (100-10 kDa; 1 C=O every 7 RU (7 mg, 0.0007 mmol)), AcOH at 10% (2 μL; 0.003 mmol) and *tb*NC (4 μL; 0.06 mmol) reacted in PBS pH = 7.0 following the general procedure described above for the Ugi-conjugation of carbonyl containing CPs. The purified conjugate was characterized by SE-HPLC (Superose® 12: conjugate rt. =14.11 min, 97.2 %; non-conjugated protein rt. = 21.99 min, 2.8 %) and **^{1}H-NMR (400 MHz, D_2O)** δ (ppm) = 5.65 (N*H*; →3)-β-L-Rha*p*OAc-(1→); 5.23 - 4.36 (anomeric C*H*s); 3.22 (C-polysaccharide (P-Cho)) 2.14 (CH_3; OAc; →3)-β-L-Rha*p*OAc-(1→); 2.03 (CH_3; N*H*Ac; α-D-Glc*p*NAc-(1→); 1.41 (CH_3; →3)-β-L-Rha*p*OAc-(1→); 1.33 (CH_3; →2)-α-L-Rhap-(1→). (**Figure S6**).

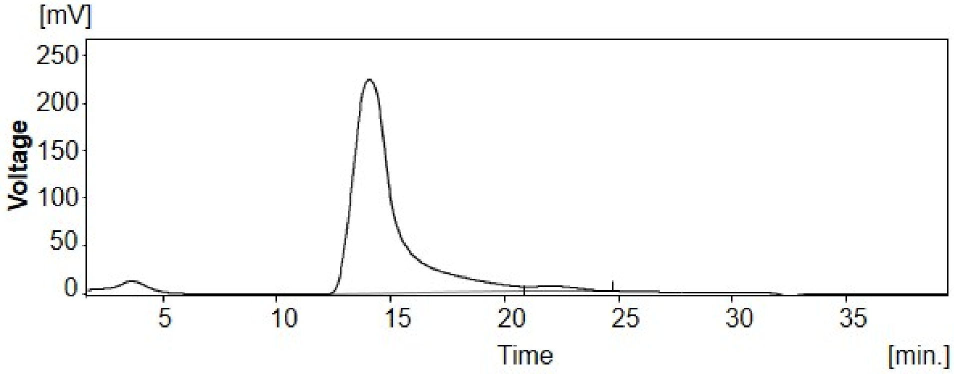

Figure S14. SE-HPLC traces (Superose® 12) of purified conjugate CPs7F-TT[a]

Synthesis of the conjugate CPs7F-DT[a]

DT[a] (10 mg, 0.0002 mmol), fragmented and oxidized CPs7F from *Streptococcus pneumoniae* (100-10 kDa; 1 C=O every 7 RU (10 mg, 0.001 mmol)), AcOH at 10 % (2 μL, 0.003 mmol) and *tb*NC (4 μL, 0.06 mmol) reacted in PBS pH = 7.0 following the general procedure described above for the Ugi-conjugation of carbonyl containing CPs. The purified conjugate was characterized by SE-HPLC (Superose® 12: conjugate rt. =14.40 min, 90 %; non-conjugated protein rt. = 10 min, 12.5 %).

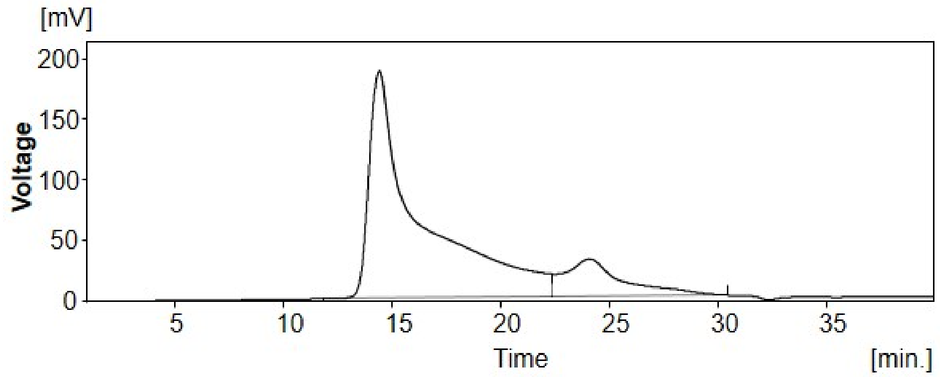

Figure S15. SE-HPLC traces (Superose® 12) of purified conjugate CPs7F-DT[a]

Synthesis of the conjugate CPs9V-TT[a]

TT[a] (5 mg, 0.00003 mmol), fragmented and oxidized CPs9V from *Streptococcus pneumoniae* (100-10 kDa; 1 C=O every 7 RU (5 mg, 0.0005 mmol)), AcOH at 10 % (2 µL, 0.003 mmol) and *tb*NC (4 µL, 0.06 mmol) reacted in PBS pH = 7.0 following the general procedure described above for the Ugi-conjugation of carbonyl containing CPs. The purified conjugate was characterized by SE-HPLC (Superose® 12: conjugate rt. = 13.77 min, 86 %; non-conjugate protein rt. = 21.71 min, 14 %) and **^{1}H-NMR (400 MHz, D_2O)** δ (ppm) = 5.51 (anomeric C*H*, →4)-α-D-Glc*p*-(1→); 5.35 (anomeric C*H*, →3)-α-D-Gal*p*-(1→); 5.08 (anomeric C*H*, →4)-α-D-Glc*p*A-(1→); 4.88 (anomeric C*H,* →3)-β-D-Man*p*NAc-(1→); 4.59 (anomeric C*H*, →4)-β-D-Glc*p*-(1→); 3.18 (C-polysaccharide (P-Cho)); 2.08 (CH_3, N*H*Ac, →3)-β-D-Man*p*NAc-(1→) (**Figure S7**).

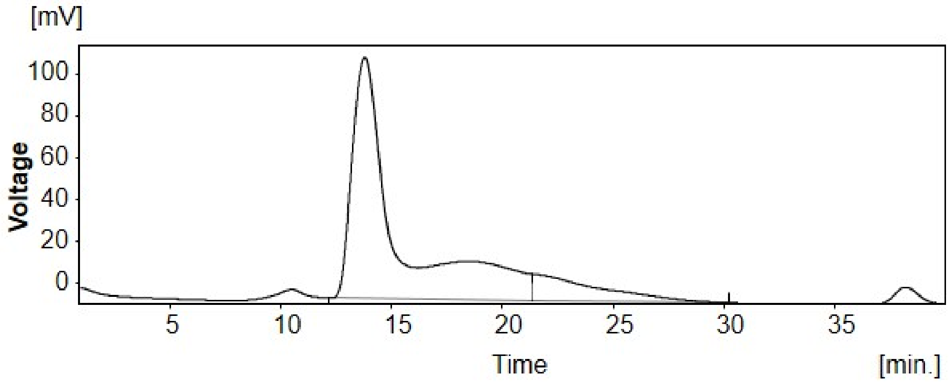

Figure S16. SE-HPLC traces (Superose® 12) of purified conjugate CPs9V-TT[a]

Synthesis of the conjugate dextran-BSA[a]

Hydrazine-activated bovine serum albumin (BSA[a]) (5 mg, 0.00007 mmol), fragmented and oxidized dextran (70 kDa; 1 C=O every 3 RU (10 mg, 0.0001 mmol), a solution at 10 % (2 µL, 0.003 mmol) and *tb*NC (2 µL, 0.03 mmol) reacted in PBS pH = 7.0

following the general procedure described above for the Ugi-conjugation of carbonyl containing CPs. The purified conjugate was characterized by SE-HPLC (Superose® 12: conjugate rt. = 14.24 min, 94.6 %; non-conjugated protein rt. = 20.40 min, 5.4 %).

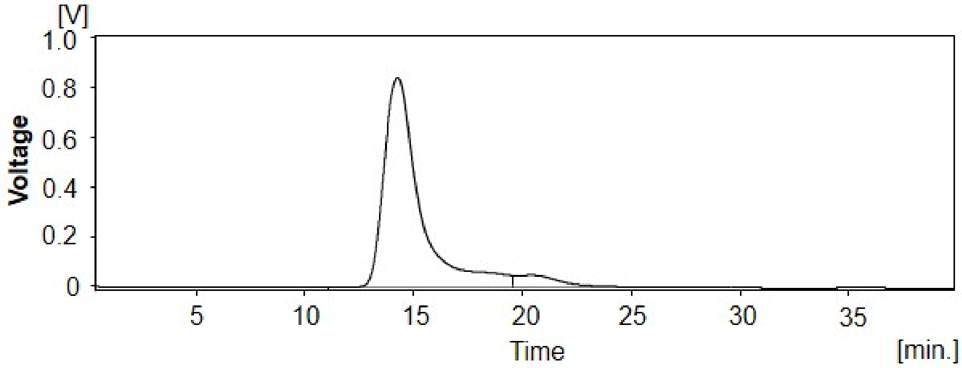

Figure S17. SE-HPLC traces (Superose® 12) of purified conjugate dextran-BSA[a]

Synthesis of the conjugate CPsVi-TT[a]

TT[a] (30 mg, 0.0002 mmol), acetone (200 µL, excess) natural CPsVi from *Samonella enterica* serovar Typhi (10 mg), and *tb*NC (4 µL, 0.06 mmol) reacted in PBS pH = 7.4 following the general procedure for the conjugation of CPsVi by Ugi-4CR. The conjugate was analyzed and purified by SE-HPLC Superose® 6, affording three different fractions (1st (50-95 min), 2nd (150-250 min) and 3rd (150-250 min)) that were analyzed by SE-HPLC Superose® 12: 1st (rt. = 15.097 min, 100 %); 2nd (rt. = 15.367 min, 100 %); 3rd (rt. = 15.560 min, 28,3 %; rt. = 23.267 min, 71.7 %). The second fraction was analyzed by SDS-PAGE (**Figure S4 E)**.

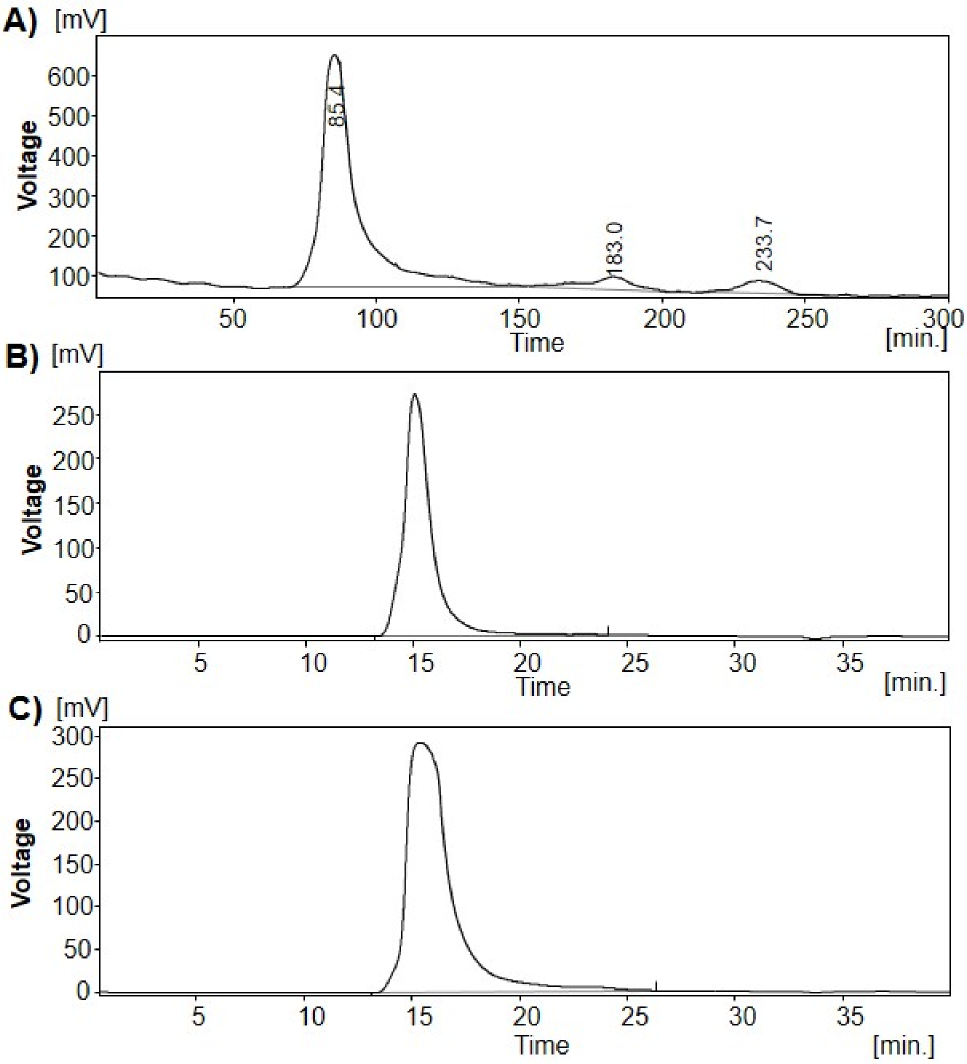

Figure S18. SE-HPLC traces of A) crude conjugate CPsVi-TT^a (Superose® 6); B) 1st fraction of purified conjugate Vi-TT^a (Superose® 12) and C) 2nd fraction of purified conjugate Vi-TT^a (Superose® 12).

Synthesis of the conjugate CPsVi-DT^a

DT^a (5 mg, 0.0002 mmol), acetone (200 μL, excess) natural CPsVi from *Samonella enterica* serovar Typhi (5 mg), and *tb*NC (4 μL, 0.06 mmol) reacted in PBS pH = 7.4 following the general procedure for the conjugation of the polysaccharide Vi by Ugi-4CR. The purified conjugate was characterized by SE-HPLC (Superose® 12: conjugate rt. = 15.3 min, 100 %) and **^{1}H-NMR (400 MHz, D_2O):** δ (ppm) = 5.06 (anomeric C*H,* GalA); 4.70 (C*H*-5, GalA); 4.47 (C*H*-4, GalA); 4.24 (C*H*-2, GalA); 4.12 (C*H*-3, GalA); 2.10 (CH_3, N*H*Ac) (**Figure S8**).

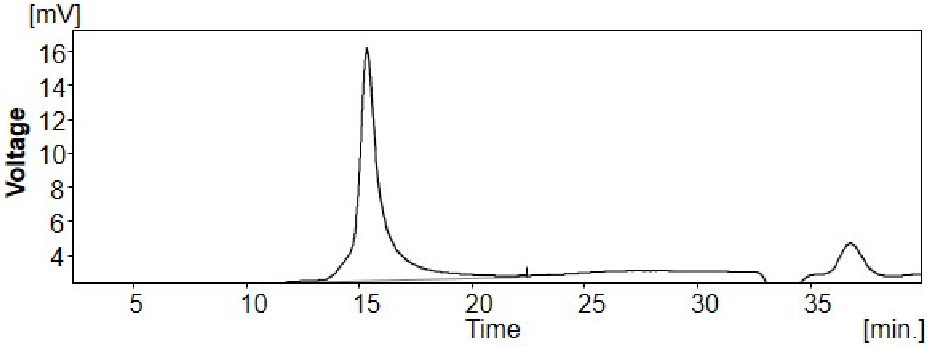

Figure S19. SE-HPLC traces (Superose® 12) of purified conjugate CPsVi-DT[a].

Synthesis of the conjugate CPs14-TT[a]-CPs7F

TT[a] (2 mg, 0.000009 mmol), fragmented and oxidized CPs/F from *Streptococcus pneumoniae* (100-10 kDa; 1 C=O every 7 RU (3 mg, 0.0002 mmol)), TEMPO-oxidized CPs14 (100-10 kDa; 1 -COOH every 2.8 RU (3 mg, 0.0002 mmol)) and *tb*NC (4 µL, 0.06 mmol) reacted overnight in PBS pH = 7.4. The conjugate was purified by diafiltration through a regenerated cellulose membrane (100 kDa) with 10 water volumes, and 2 bar nitrogen pressure. The purified conjugate was characterized by SE-HPLC (Superose® 12: conjugate rt. = 14.4 min, 100 %) and **^{1}H-NMR (500 MHz, D_2O)** δ (ppm) = 5.64 [CPs7F (N*H*; →3)-β-L-Rha*p*OAc-(1→)]; 5.23 – 4.35 [CPs7F + CPs14 (anomeric C*H*s)]; 3.23 (C-polysaccharide (P-Cho)); 2.14 [CPs7F (CH_3; OAc; →3)-β-L-Rha*p*OAc-(1→)]; 2.04 [CPs14 (CH_3, N*H*Ac, →4)-β-D-GlcNAc-(1→)]; 2.03 [CPs7F (CH_3; N*H*Ac; α-D-Glc*p*NAc-(1→)]; 1.41 (CH_3; →3)-β-L-Rha*p*OAc-(1→); 1.33 (CH_3; →2)-α-L-Rhap-(1→). (**Figure S9**)

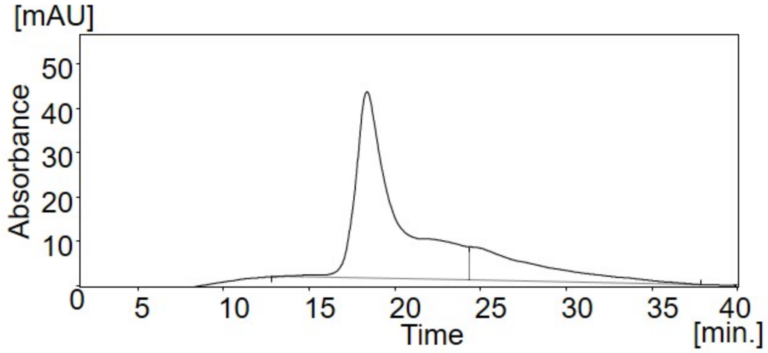

Figure S20. SE-HPLC traces (Superose® 12) of purified conjugate CPs14-TT[a]-7F.

Colorimetric determinations

Sulfuric acid-orcinol method

Seven standard solutions containing anhydrous D (+) glucose with concentrations 0.05; 0.02; 0.10; 0.20; 0.40; 0.60; 0.80 mg/mL were prepared from a solution of 1 mg/ mL to construct a calibration curve. 100 µL were took from the different samples, the standard solution and the blank. 200 µL of a solution containing 2 % of orcinol and 25 % of H_2SO_4 (v/v) were added and the mixtures were stirred vigorously. Then, 1.5 mL of a solution containing 60 % of H_2SO_4 (v/v) were added. After stirring vigorously, the mixtures were heated up to 80 ^oC for 20 min and then the absorbance was read at 530 nm. A gravimetric factor of 1.7 was used for CPs7F due to its structure contains a monosaccharide that does not reveal with this method.

Biphenyl method

A calibration curve was constructed preparing solutions of galactouronic acid (98 %, Fluka) from 1 to 20 µg/mL. 200 µL from the samples, the standard solutions and the blank were took, the solutions were cooled to 0 ^oC and then 1.2 mL of a sodium borate solution (98 %, Merck) in H_2SO_4 were added. The mixtures were stirred and heated up to 100 ^oC for 5 min in a water bath. Then, they were cooled in an ice bath and 20 µL of a solution containing 0.1 % of biphenyl (90 %, Aldrich) and 0.5 % of NaOH were added to the samples and the standards, while 20 µL of a solution containing 0.5 % of NaOH were added to the blank. The mixtures were stirred and incubated at room temperature for 5 min and then, pink colored samples were read at 520 nm. The concentration was calculating having a count of 4.35 as gravimetric factor.

Lowry's method

Six standard solutions containing BSA with concentrations 10; 20; 40; 60; 80; 100 µg/mL were prepared from a solution of BSA at 1 mg/mL to construct a calibration curve. 200 µL were took from the samples, the standard solutions and the blank, 100µL of solution containing NaOH (1 M) were added and the mixtures were stirred vigorously. After 10 min at room temperature, 100 µL of a solution containing 1 mol/L of Follin Ciocalteau reactant were added and then the mixture was stirred vigorously. The mixtures were incubated at room temperature for 30 min and then their absorbance was read at 750 nm.

Hestrin's method

Five standard solutions containing acetylcholine with concentrations 0.05; 0.125; 0.250; 0.375; 0.750 μmol/mL were prepared from a solution of acetylcholine at 3.3 μmol/mL to construct a calibration curve. 1500 μL were took from the sample, the standard solutions and the blank. 250 μL of a solution containing HCl: H_2O (1:2) (v/v) were added to the blank and the mixture was stirred. Then, 500 μL of a solution containing hidroxylamine (2 M) and 500 μL of a solution containing NaOH (3.7 M) were added to the samples, the standard solutions and the blank, and the mixtures were stirred vigorously. After 4 min, 250 μL of a solution containing HCl: H_2O (1:2) (v/v) were added to all the tubes except for that containing the blank and they were stirred vigorously. 250 μL of a solution containing $FeCl_3$ (0.74 mM) in HCl (0.2 M) were added to all the tubes and the mixtures were stirred vigorously. Their absorbance was read at 540 nm.

Electrophoresis on polyacrylamide gel (SDS-PAGE)

In the case of glycoconjugates containing TT as carrier protein a separating gel with 7 % of polyacrylamide was used, while in the case of glycoconjugates containing DT a a separating gel with 10 % of polyacrylamide was used. In all cases a resolving gel with 4 % of polyacrylamide was used. All the samples were dissolved in a Laemmli buffer and heated in a water bath at 100 ^{o}C for 5 min. The experiments were run at 250 mV. In order to identify the protein, 10 μg of each conjugate and 10 μg of the control protein (TT or DT) were applied and a Coomassie staining protocol was used.[10] In order to identify the carbohydrate, 20 μg of each conjugate and 20 μg of the control protein (TT or DT) were applied and a Fuchsin staining protocol was used. The gels were incubated in a solution containing acetic acid: methanol: water (10:35:55) for 2h at room temperature. Then, the gels were protected from light and incubated for 1 h in a solution containing 0.78 % of periodic acid and 5 % of acetic acid. The gels were washed twice with a solution containing 0.2% of $Na_2S_2O_5$ for 5 to 10 min until they were colorless. A basic Fuschin solution was added and maintained for a period of 1 to 2 h until the gels developed red coloration. At the end, the gels were washed with a solution containing acetic acid: methanol: water (10:35:55) and those lanes containing carbohydrates maintained the red coloration.

Immunization schemes

Female Balb/c mice within 5 to 6 weeks old and between 18 and 20 g of weight (National Center for the Production of Laboratory Animals (CENPALAB)) were used. The mice were adapted to the laboratory conditions in Macrolon boxes (Panlab, Spain) during 7 days: under controlled humidity and temperature (22-24 ^{o}C), with 12 h of light and 12 h of darkness. Groups of 10 Balb/c mice were immunized at 0, 14 and 28 days by subcutaneous route and blood samples were collected by the retro-orbital plexus 3 days before starting the assay and 7 days after each immunization. Doses with 2 µg of carbohydrate content from conjugates (CPs14-TT^{a}, CPs14-DT^{a}, CPs7F-TT^{a}, CPs7F-DT^{a}, CPs9V-TT^{a} and CPs14-TT^{a}-CPs7F) were administered in each immunization, while the control group, immunized with CPs 7F, 14, 9V, received 25 µg/doses of CPs. Moreover, a placebo group was included, being immunized with $AlPO_4$, at 125 µg/dose of elemental aluminum. All immunogens were also adsorbed on $AlPO_4$, at 125 µg/dose of elemental aluminum ending with a total volume of 0.1 mL. The extracted blood samples were centrifuged at 6000 rpm (Sigma 3-18K) during 5 min and the sera were conserved at -20 ^{o}C until the moment of the immunologic evaluation. The experimental protocols were approved by the Committee of Ethic for the Experimentation with Lab Animals and the Quality Department of the Finlay Institute of Vaccines.

In the case of the conjugates Vi-DT and Vi-TT, female Balb/c mice within 5 to 6 weeks old and between 16 and 18 g of weight (National Center for the Production of Laboratory Animals (CENPALAB)) were used. The mice were adapted to the laboratory conditions in Macrolon boxes (Panlab, Spain) during 7 days: under controlled humidity and temperature (23-27 ^{o}C), with 12 h of light and 12 h of darkness. Groups of 10 Balb/c mice were immunized at 0, 14 and 28 days by subcutaneous way and blood samples were collected by the retro-orbital plexus 3 days before starting the assay and 7 days after each immunization. Doses with 2.5 µg of carbohydrate content from conjugates (Vi-TT^{a} and Vi-DT^{a}) were administered in each immunization, while the control group, immunized with CPsVi received 2.5 µg/doses of CPs. Moreover, a placebo group was included, being immunized with PBS. The extracted blood samples were incubated at 37 ^{o}C for 30 min, then cooled to -20 ^{o}C, centrifuged at 1500 rpm (Sigma 3-18K) during 5 min and the sera were conserved at -20 ^{o}C until the moment of the immunologic evaluation.

ELISA analysis

Determination of the antibodies (IgG) against the CPs

The determination of IgG titers induced by the conjugates against CPs serotypes 14, 7F and 9V from *Streptococcus pneumoniae* and CPsVi from *Salmonella enterica* serovar Typhi was made in polystyrene 96 well microtiter plates (Maxisorp, NUNC, Denmark). In the case of the CPs serotypes 14, 7F and 9V from *S. pneumoniae,* the plates were coated with natural CPs at a concentration of 10 µg/mL in PBS pH= 7.2 (50 µL/well) and incubated overnight at 4 ^{o}C. In the case of the CPsVi from *S.* Typhi, the plates were coated first with poli-D-Lysin and incubated at 4°C, then they were coated with the natural CPs at a concentration of 10 µg/mL in PBS pH= 7.2 (50 µL/well) and incubated overnight All plates were washed with a solution of PBS and 0.05 % tween$_{20}$. Then a blocking solution of 2 % BSA in PBS pH = 7.2 (150 µL/well) was applied and the plates were incubated at 37 ^{o}C for 30 min. Sera from the immunized mice were applied (100 µL/well) in serial dilutions (1/2) starting from 1:100 in a buffer (PBS pH = 7.2, 0.05 % Tween$_{20}$, EDTA 0.01 M, BSA 1 %) and the plates were incubated for 90 min at room temperature. Anti-Mouse IgG (whole molecule) serum conjugated to peroxidase (100 µL/well) in a dilution 1: 20000) was then added and the plates were incubated for 90 min at room temperature. At the end, 100 µL/well of a solution containing the buffer Na_2HPO_4 52 mM/ citric acid 25 mM (pH= 5.6), 1 mL of the substrate 3,3',5,5'-tetramethylbenzidine (TMB), 9 mL of sodium citrate and 2 µL of H_2O_2 were added. The reactions were carried out during 20 min in the darkness and were stopped adding 50µL/well of a solution of H_2SO_4 (3 M). The optical density at 405 nm was determined using an ELISA reader (SUNRISE, Tecan, Austria) and the software Magellan 7.0. After each step the plates were washed with a washing solution (PBS pH= 7.2 / Twen$_{20}$ 0.05 %) using an automatic washer MW-2001 ProWash (TecnoSUMA international), and then dried.

The IgG titers against CPs were defined as the logarithm of the highest dilution giving two fold the absorbance value of pre-immune sera. Statistical analyses were performed using Graph Pad Prism 4.03. First was applied the Kruskal–Wallis non-parametric test and then the Dunn's multiple comparison test where $P < 0.05$ was considered statistically significant.

Determination of the antigenicity of the CPs on the glycoconjugates

Maxisorp 96 well microtiter plates were coated with the glycoconjugates containing CPs 14, 7F, 9V, 6B and 23F at a concentration of 10 µg/mL in a coating buffer containing $NaHCO_3$ (pH = 9.6) (50 µL/well) and were incubated overnight at 4 °C. Then, a blocking solution of 1 % BSA in PBS pH= 7.2 (150 µL/well) was applied and maintained for 30 min at 37 °C. Specific sera from *Statens Serum Institute*, Copenhagen: factor serum 9g (9V) (lot N9g32 A1), factor serum 7b (7F, 7A) (lot N7b11 C1), a type serum 14 (lot M1412 A114), factor serum 6c (6B) (lot M6c12E1) and factor serum 23b (23F) (lot N23b11C1) were used. All specific sera were diluted (1: 20 000) in a buffer (PBS pH= 7.2, $Tween_{20}$ 0.3 %, EDTA 0.01 M, BSA 1 %) and the plates were incubated (100 µL/well) for 90 min at room temperature. Anti-Rabbit IgG (whole molecule) serum conjugated to peroxidase (100 µL/well) in a dilution 1: 20 000) was then added and the plates were incubated for 90 min at room temperature. At the end, 100 µL/well of a solution containing the buffer Na_2HPO_4 (52 mM)/ citric acid (25 mM) (pH= 5.6), 1 mL of the substrate TMB, 9 mL of sodium citrate and 2 µL of H_2O_2 were added. The reaction was protected from light and carried out during 20 min, and was stopped adding 50 µL/well of a solution of H_2SO_4 (3M). The optical density at 405 nm was determined using an ELISA reader (SUNRISE, Tecan, Austria) and the software Magellan 7.0. After each step the plates were washed with a washing solution (PBS pH= 7.2 / $Tween_{20}$ 0.05 %) using an automatic washer MW-2001 ProWash (TecnoSUMA internacional), and then dried.

Determination of the antigenicity of fragmented CPs and OxCPs

Specific sera from *Statens Serum Institute*: factor serum 9g (9V) (lot N9g32 A1), factor serum 7b (7F, 7A) (lot N7b11 C1) and type serum 14 (lot M1412 A114) were incubated overnight at 4 °C with a wild range of concentration (500; 50; 5; 0.5; 0.05 and 0.005 µg/mL) related with CPs, fragmented Ps or OxPs. Then the mix (sera and target) were applied on a coated-CPs (10 µg/mL) and blockade-BSA plates. After incubating for 90 min at room temperature, Anti-Rabbit IgG (whole molecule) serum conjugated to peroxidase (100 µL/well) in a dilution 1: 20000) was added and the plates were incubated for 90 min at room temperature. Either, 100 µL/well of a solution containing the buffer Na_2HPO_4 (52 mM)/ citric acid (25 mM) (pH = 5.6), 1 mL of the substrate TMB, 9 mL of sodium citrate and 2 µL of H_2O_2 were applied. After incubating for 20 min in the darkness, the reaction was stopped with 50 µL/well of a solution of H_2SO_4

(3M). The optical density at 405 nm was determined using an ELISA reader (SUNRISE, Tecan, Austria) and the software Magellan 7.0. The inhibition percentage was calculated as follows: inhibition percentage= [1-(absorbance of serum with inhibitor/ absorbance of serum without inhibitor)] x 100.

Determination of the antibodies elicited by the Ugi-linkage

Maxisorp 96 well microtiter plates were coated with the Dext-BSA conjugate at a concentration of 1 μg/mL of protein content in a $NaHCO_3$ coating buffer (pH= 9.6) (50 μL/well) and were incubated overnight at 4 ^oC. Then, a blocking solution of 2 % BSA in PBS (pH = 7.2) (150 μL/well) was applied and maintained for 30 min at 37 ^oC. Sera from the third dose of the immunization with the conjugates (14-TT^a, 14-DT^a) were added in serial dilutions (1/2) starting from 1:100 in a buffer (PBS pH = 7.2, Tween$_{20}$ 0.3 %, EDTA 0.01 M, BSA 1 %). The plates (100 μL/well) were incubated for 90 min at room temperature. Anti-Mouse IgG (whole molecule) serum conjugated to peroxidase ((100 μL/well) in a dilution 1: 20000) was then added and the plates were incubated for 90 min at room temperature. At the end, 100 μL/well of a solution containing the buffer Na_2HPO_4 52 mM/ citric acid 25 mM (pH= 5.6), 1 mL of the substrate TMB, 9 mL of sodium citrate and 2 μL of H_2O_2 were added. The reaction was carried out during 20 min in the darkness and was stopped adding 50μL/well of a solution of H_2SO_4 3M. The optical density at 405 nm was determined using an ELISA reader (SUNRISE, Tecan, Austria) and the software Magellan 7.0. After each step the plates were washed with a washing solution (PBS pH= 7.2 / tween$_{20}$ 0.05 %) using an automatic washer MW-2001 ProWash (TecnoSUMA international), and then dried.

Determination of the antigenicity of the conjugate CPsVi-TT^a by dot-blot

The protein, the CPsVi and the Ugi-conjugates (5 μL) at 125 μg/mL; 250 μg/mL and 1.700 mg/mL were applied on a nitrocellulose membrane previously moistened with PBS (pH = 7.4) and incubated in a solution containing PBS (pH = 7.4) and 3 % skim milk at 37°C for 30 min. The membrane was washed 3 times (during 5 min) with a washing solution containing PBS (pH = 7.4) and tween$_{20}$ (0.05 %) and then, was incubated in a solution containing the anti-CPsVi MAb 4G3E11, PBS (pH = 7.4) and 1% skim milk. After washing the membrane 3 times (during 5 min) with the washing solution, it was incubated with a solution containing anti-IgG (in a dilution 1:5000), PBS (pH = 7.4) and 1% skim milk. Finally, the membrane was washed 3 times (during

10 min) with the washing solution and revealed using diaminobenzidine hydrochloride (DAB) and hydrogen peroxide.

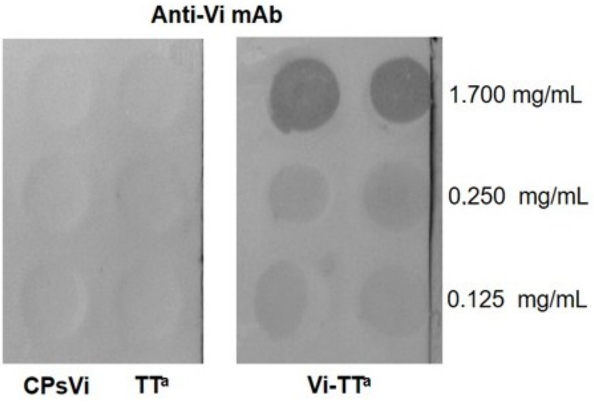

Figure S21. Dot-blot experiment of conjugate Vi-TT^a in the presence of an anti-Vi monoclonal antibody. CPsVi and TT^a were used as control.

Opsonophagocytic assays (OPA)

The OPA assays were designed based on that described by Romero-Steiner in 1997, using human polymorphonuclear leukocytes as effector cells. Heat inactivated mouse sera (pool of CPs14-TT^a, CPs7F-TT^a and CPs14-TT^a-Cps7F of last immunization bleed sera of 10 mice) and were serially diluted in twofold steps in a 96-well microtiter plate (round bottom, COSTAR) with opsono buffer (Hank´s buffer (Sigma) containing 0.1 % gelatin and were incubated with cells of *S. pneumoniae* serotypes (~1000 CFU per well) during 15 min at 37 ^oC in a 5 % CO_2 atmosphere. Complement from baby rabbit serum (Pel-Freez Biological) and peripheral polymorphonuclear leukocytes purified from human blood were added at 4×105 cell per well. The mixture was incubated at 37 ^oC for 45 min with shaking (90 rpm). The reaction was stopped with ice-cooling during 5 min. After the phagocytic step, an aliquot of 5 µL was extracted and applied onto an agar plate covered with a tilted Todd Hewitt yeast extract, and the plate was incubated at 37 ^oC with 5 % CO_2 atmosphere for 18 h. OPA titters were calculated as the reciprocal of the sera dilution that caused a 50 % reduction of the CFU (killing) compared with the CFU from the control wells containing all reagents except for the mouse sera.

References

1. C. Jones, *J. Pharmaceut. Biomed.*, 2005, **38**, 840-850.
2. M. Moreau, J. C. Richards and M. B. Perry, *Carbohydr. Res.*, 1988, **182**, 79-99.

3. S. van Selm, M. A. B. Kolkman, B. A. M. van der Zeijst, K. A. Zwaagstra, W. Gaastra, J. P. M. van Putten, *Microbiology*, 2002, **148**, 1747-1755.
4. O. H. Lowry, N. J. Rosebrough, A. L. Farr and R. J. Randall, *J. Biol. Chem.*, 1951, **193**, 265-275.
5. J. Bruckner, *Biochem.*, 1954, **60**, 200-205.
6. T. M. Filisetti-Cozzi, N. C. Carpita, *Anal. Biochem.*, 1991, **197**, 157-162.
7. S. Hestrin, *J. Biol. Chem.*, 1949, **180**, 249-261.
8. WHO Technical Report Series, *Requirements for Diphteria, Tetanus, Pertussis and Combined Vaccines*, 1990.
9. WHO Technical Report Series, *Recommendations for Diphtheria, tetanus, pertussis and combined vaccines, Amendments 2003*, 2003.
10. S. F. D. S. Groth, R. G. Webster, A. Datyner, *Biochim. Biophys. Acta* 1963, **71**, 377-391.
